# Subdiffusion from competition between multi-exponential friction memory and energy barriers

**DOI:** 10.1140/epje/s10189-025-00518-y

**Published:** 2025-09-12

**Authors:** Anton Klimek, Benjamin A. Dalton, Roland R. Netz

**Affiliations:** Fachbereich Physik, Freie Universität Berlin, Arnimalle 14, 14195 Berlin, Germany

## Abstract

**Abstract:**

Subdiffusion is a hallmark of complex systems, ranging from protein folding to transport in viscoelastic media. However, despite its pervasiveness, the mechanistic origins of subdiffusion remain contested. Here, we analyze both Markovian and non-Markovian dynamics, in the presence and absence of energy barriers, in order to disentangle the distinct contributions of memory-dependent friction and energy barriers to the emergence of subdiffusive behavior. Focusing on the mean squared displacement (MSD), we develop an analytical framework that connects subdiffusion to multi-scale memory effects in the generalized Langevin equation (GLE), and derive the subdiffusive scaling behavior of the MSD for systems governed by multi-exponential memory kernels. We identify persistence and relaxation timescales that delineate dynamical regimes in which subdiffusion arises from either memory or energy barrier effects. By comparing analytical predictions with simulations, we confirm that memory dominates the overdamped dynamics for barrier heights up to approximately $2\,k_BT$, a regime recently shown to be relevant for fast-folding proteins. Overall, our results advance the theoretical understanding of anomalous diffusion and provide practical tools that are broadly applicable to fields as diverse as molecular biophysics, polymer physics, and active matter systems.

**Graphical abstract:**

Subdiffusion in the context of the generalized Langevin equation can arise due to energy barriers, from friction memory or from a combination of both. We derive the power-law scaling for multi-exponential memory functions that directly translates to the subdiffusive scaling in the MSD. This allows us to disentangle contributions from energy barriers and from memory. It turns out that memory governs the subdiffusion for small energy barriers in the order of a few $k_BT$. For higher energy barriers, the short time dynamics are still dominated by memory and long-time dynamics are governed by a combination of memory effects and energy barrier contributions.
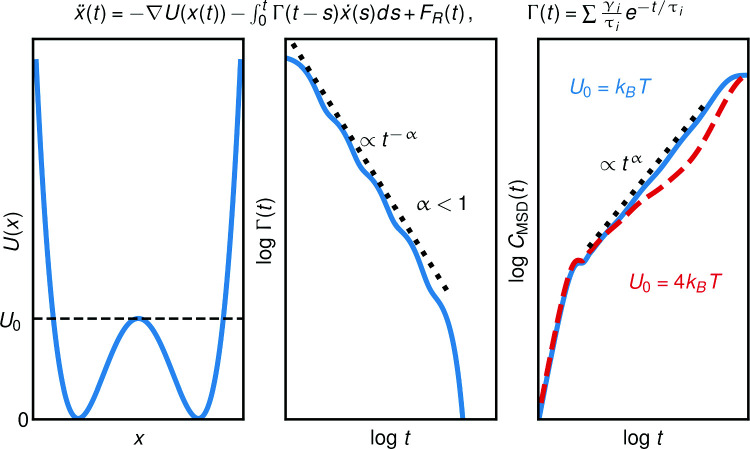

**Supplementary Information:**

The online version contains supplementary material available at 10.1140/epje/s10189-025-00518-y.

## Introduction

Diffusion is a fundamental transport phenomenon observed across a wide range of physical, chemical, and biological systems. It is driven by interactions of an observable with its fluctuating environment, which results in random motion. Therefore, the dynamics of a single diffusing observable are described by a random walk and its statistics are captured by ensemble or time averages, which are equivalent for stationary systems [1]. An important statistical description of the dynamics of a system is the MSD, defined as $C_{\text {MSD}}(t) = \langle (x(0) - x(t))^2 \rangle$, where $\langle \cdot \rangle$ denotes an ensemble average and *x* is the observable of interest. $C_{\text {MSD}}(t)$ characterizes the diffusion dynamics of an observable and can reveal important information about the physical properties of the environment. In general, the MSD can be written in the form $C_\mathrm{{MSD}}(t)\propto t^{\alpha (t)}$ with the time-dependent exponent $\alpha (t)$ describing the mode of diffusion at a given time. The most common model of diffusion is characterized by a linear increase in the MSD in time, $\alpha (t)=1$, and is referred to as Brownian- or normal diffusion. Brownian diffusion can describe the dynamics of a molecule in a liquid [2], but also the dynamics of stock prices [3] or the motion of certain cells [4]. However, the dynamics of many physical, chemical, and biological systems are known to deviate from normal diffusive behavior [5–8]. The mode of diffusion of an observable is characterized by the time-dependent exponent, given by

1 $$\begin{aligned} \alpha (t) = \frac{{d} \ln \big (C_{\text {MSD}}(t)\big )}{{d} \ln (t)}\,, \end{aligned}$$

where $\alpha (t)<1$ is called subdiffusion, $\alpha (t)>1$ superdiffusion, the special case $\alpha (t)=1$ defines normal diffusion and $\alpha (t)=2$ defines ballistic motion. It is often the case that for a given observable, $\alpha (t)$ is not constant but rather varies significantly as a function of *t*. As such, systems often exhibit different modes of diffusion on different time scales.

Examples of superdiffusion include the motion of animals [9], active particles [10] and actively transported particles within cells [11] and are often related to non-equilibrium phenomena. Subdiffusion, on the other hand, appears in crowded, caged or viscoelastic environments and for systems with energy barriers in the potential landscape. Examples of subdiffusion include cell migration [12, 13], polymer network dynamics [14, 15], biomolecule diffusion in crowded environments [16–18], protein folding dynamics [19–21] and many more. The interplay between energy barriers and viscoelastic effects, that both contribute to deviations from normal diffusion, is still not well understood.

Theoretical approaches to describe subdiffusive phenomena use diverse models such as correlated time random walks [22–24] or fractal Brownian motion [25, 26]. Some of these models are special cases of the generalized Langevin equation (GLE), which is derived exactly from a many-body Hamiltonian via projection [27–29]. The GLE incorporates non-Markovian effects, i.e., a trajectory’s memory of its past, which naturally arises when projecting the dynamics of a many-body system on a low- or one-dimensional observable of interest [27, 28, 30].

The GLE describes the motion of a general observable *x* in a complex environment as

2 $$\begin{aligned} \ddot{x}(t)=-\nabla U(x(t)) -\int _{0}^{t} \Gamma (t-t')\dot{x}(t') dt' +F_R(t) \,, \end{aligned}$$

where $\Gamma (t)$ is the friction memory kernel that describes the dependence of the friction force on previous velocities, $-\nabla U(x)$ is the force due to a potential, and $F_R(t)$ is a random force that incorporates the fluctuations of the environment. Note that no mass appears in front of the acceleration term, as is appropriate for a general description including equilibrium and non-equilibrium systems, so that all terms in Eq. (2) have units of acceleration. The correlation of the random forces using the Mori projection in a harmonic potential *U*(*x*) [27] fulfills the relation

3 $$\begin{aligned} \langle F_R(0) F_R(t) \rangle = B \Gamma (t)\,, \end{aligned}$$

where $B=\langle \dot{x}^2\rangle$, which holds only approximately for non-harmonic potentials *U*(*x*) [28, 31]. Often, the observable mass *m* is defined via an analog of the equipartition theorem as $B=k_{\text {B}}T/m$, where $k_{\text {B}}$ is the Boltzmann constant and *T* denotes the temperature of the observable’s environment. Here, we decide to keep the more general notation and use *B*. A GLE Eq. (2) can also be derived for non-equilibrium systems [32] with an expression for the random force fluctuations that is more general than Eq. (3). Such non-equilibrium GLEs can be used to describe cell motion [33–35] or other actively driven processes [15].

Models that describe subdiffusive phenomena within the GLE framework often assume power-law memory $\Gamma (t)\propto t^{-\alpha }$ motivated by fractal patterns and self-similarity of the systems under consideration [36–38]. It was shown that power-law memory can efficiently be modeled by sums of exponential memory components [39, 40]. In fact, protein folding dynamics observed in molecular dynamics (MD) simulations have been described using multi-exponential memory, which accurately captures the subdiffusive behavior in protein dynamics [41, 42].

In this paper, we investigate how multi-exponential memory gives rise to subdiffusivity, characterized by a scaling exponent ${\alpha < 1}$ in the MSD. We introduce an analytical framework for predicting the MSD for non-Markovian systems with multi-exponential memory kernels, which is exactly solvable for free diffusion and diffusion in a harmonic potential. We compare the analytical results with non-Markovian simulations in a double-well potential to determine the respective roles of memory-dependent friction and energy barriers in determining subdiffusive dynamics. Motivated by the exponential memory observed in protein folding [41–43], we use multi-exponential memory with exponentially spaced memory times and amplitudes and derive an exact expression for the scaling exponent $\alpha$ of the MSD. We find that memory dictates the dynamics on short and intermediate time scales, and we give expressions for the time scales on which memory effects dominate over energy barriers in determining the MSD. We determine the transition time between ballistic and subdiffusive behavior and show that the ballistic regime of the MSD is extended under the influence of memory. Furthermore, we show that for small energy barriers (on the order of a few $k_\text {B}T$), the influence of the potential landscape on the MSD is negligible in comparison with the influence of memory effects, until reaching time scales at which the system probes the global spatial confinement that is present for every spatially bounded potential. Overall, our results shed light on the connection between multi-scale memory and subdiffusion, and we demonstrate that under many conditions, memory effects are more important than the potential landscape in generating subdiffusive dynamics.

## Non-Markovian diffusion model

**Fig. 1:**
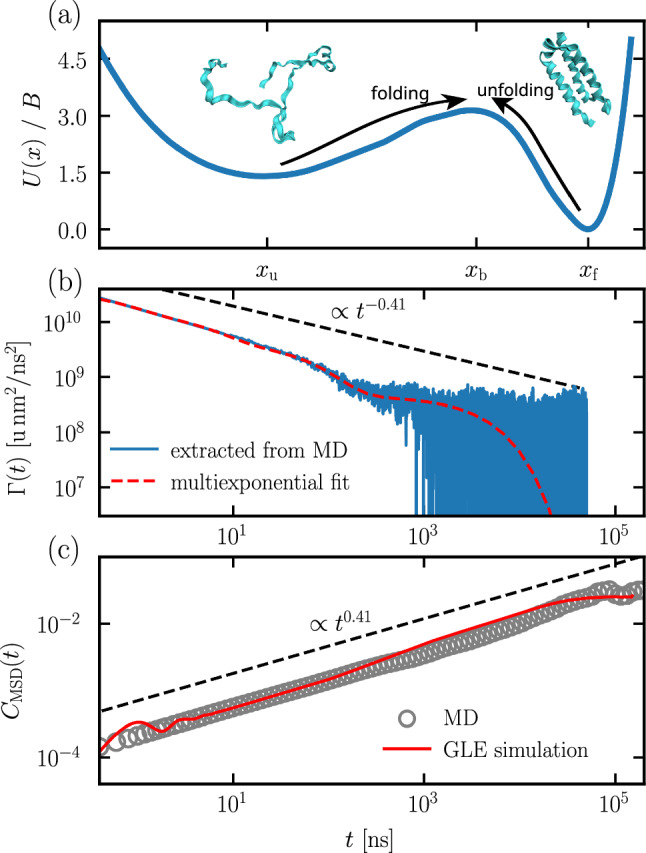
**a** Potential *U*(*x*) extracted from MD simulations of the fast-folding protein $\alpha _3$D, originally published in [44], as a function of the fraction of native contacts reaction coordinate *x*. $x_\mathrm{{f}}=0.75$ and $x_\mathrm{{u}}=0.52$ are the locations of the folded and unfolded states, respectively, and $x_\mathrm{{b}}=0.67$ is the location of the barrier top. Representative structures for the folded and unfolded states are shown at their respective positions. **b** Friction kernel $\Gamma (t)$ of the fraction of native contacts reaction coordinate for $\alpha _3$D, extracted from MD trajectories, and the corresponding multi-exponential fit of Eq. (4) to the extracted kernel. **c** MSD of the fraction of native contacts reaction coordinate of $\alpha _3$D from MD (gray circles). The red line shows results for the simulation of the GLE (Eq. (2)) with the fitted friction kernel $\Gamma (t)$ in (**b**) and the potential *U*(*x*) in (**a**). The dashed lines in (**b**) and (**c**) display pure power-law behavior with $\alpha$ determined by a logarithmic time average of Eq.(1), i.e., an average using exponentially spaced time points, over the MD data shown in (**c**)

The friction memory kernel $\Gamma (t)$, which represents dissipative interactions with a dynamic environment, can take different forms depending on the specific details of a particular system. In the context of subdiffusive phenomena, two types of kernels are often considered: power-law kernels [45, 46] and sums of exponentials [40, 41, 47]. Power-law kernels can be derived from Hamiltonian models that contain infinitely many harmonically coupled heat-bath particles coupled to the observable of interest. These models have been amply studied theoretically [48]. Power-law kernels result in subdiffusive behavior across all time scales. However, relaxation times of physical systems are typically bound by both a smallest and a largest time scale. It has been shown that the dynamics for systems with multi-exponential memory kernels can give rise to power-law behavior in the corresponding MSDs over many orders of magnitude, with short-time and long-time cutoffs [39, 40]. In fact, the dynamics of many fast-folding proteins are well described by a GLE with multi-exponential memory kernels, moving over a potential landscape *U*(*x*) [41–43, 49]. To illustrate this, in Fig. 1a, we show the potential of mean force extracted from all-atom simulations of the fast-folding protein $\alpha _3$D, and in Fig. 1b we show that the multi-exponential memory kernel $\Gamma (t)$ extracted from molecular dynamics simulations of $\alpha _3$D [42, 44] closely resembles a power-law kernel below an upper cutoff time. A GLE simulation can be parameterized using the multi-exponential fit to the memory kernel shown in Fig. 1b and the extracted potential *U*(*x*) shown in Fig. 1a. The time-averaged MSD of a finite trajectory in a potential depends on the starting position. Therefore, we perform an additional ensemble average over 1000 independent simulation trajectories, where initial conditions are drawn from the according Boltzmann distribution to obtain the initial-position-independent MSD from our GLE simulation. The MSD generated by the GLE simulation can then be compared to the MSD directly extracted from the all-atom MD simulations. In Fig. 1c, we show that the GLE with multi-exponential memory accurately reproduces the MD simulation MSD. Therefore, in this paper, we consider multi-exponential kernels of the form

4 $$\begin{aligned} \Gamma (t) = \sum _{i=1}^{n}\frac{\gamma _i}{\tau _i} e^{-t/\tau _i} \,. \end{aligned}$$

The potential *U*(*x*) in Fig. 1a exhibits two distinct local minima corresponding to the folded and unfolded states, separated by a barrier of height $U_0=3B$ from the folded state (corresponding to a barrier energy of $3\,k_{\text {B}}T$), where the observable *x* is the fraction of native contacts reaction coordinate [42, 50], commonly used for describing the conformational states in protein simulations. Energy barriers, such as those observed in Fig. 1a, occur in many two- or multi-state systems. How such energy barriers influence the MSDs that are measured in dynamic systems and what role the barriers play in comparison to friction memory effects, is still an open question. Recent investigations into protein folding dynamics [41, 42] and into the role of power-law kernels fitted by exponentials over finite time intervals [39, 40, 51] demonstrate the importance of exponential memory contributions in complex systems. Motivated by the applicability to protein folding and other complex systems, and aiming to systematically analyze the dynamics of observables that exhibit multi-exponential friction memory kernels, we consider exponentially spaced memory-time scales and friction amplitudes, according to

5 $$\begin{aligned} \begin{aligned} \tau _i&= \tau _1 c^{i-1} \\ \gamma _i&= \gamma _1 d^{i-1}\,, \end{aligned} \end{aligned}$$

where *c* determines the memory-time ratio $\tau _{i+1}/\tau _i$ and *d* determines the friction-amplitude ratio $\gamma _{i+1}/\gamma _i$.

In SI Sec. I, we derive the scaling exponent $\alpha$ of the MSD for a multi-exponential memory kernel defined by Eqs. (4), (5) as

6 $$\begin{aligned} \alpha (c,d) = \ln (c/d) / \ln (c), \end{aligned}$$

valid for $c \gg d >1$ and for times $\tau _1<t<\tau _n$. We verify Eq. (6) further below by comparison with our analytic and simulation results for the MSD.

It is useful to define the running integral of the friction kernel as

7 $$\begin{aligned} G(t) = \int _{0}^{t} \Gamma (s) ds\,. \end{aligned}$$

From this, the inertial time is defined as

8 $$\begin{aligned} \tau _m = \frac{1}{G(\infty )} = \frac{1}{\sum _{i=1}^n \gamma _i}\,, \end{aligned}$$

which governs the long-time diffusivity via $D=B\tau _m$. In the Markovian limit, where all memory times are small compared to the inertial time ($\tau _i \ll \tau _m$), one recovers the Markovian Langevin equation [52, 53] given by

9 $$\begin{aligned} \ddot{x}(t) = -\nabla U(x(t)) - \frac{1}{\tau _m}\dot{x}(t) + F_R(t)\,, \end{aligned}$$

which leads to dynamics with a short-time ballistic regime in the MSD with $\alpha =2$, directly followed by long-time diffusive motion with $\alpha =1$. In the case of free diffusion ($U(x)=0$), the diffusive regime continues unbounded, whereas potentials of finite width lead to confinement, with a long-time plateau in the MSD and correspondingly $\alpha =0$ for long times. The timescale at which the ballistic regime terminates defines the persistence time $\tau _p$, which is $\tau _p=\tau _m$ in the Markovian case with $\tau _i \ll \tau _m$. As we discuss below, in the presence of longer memory times $\tau _i > \tau _m$, the persistence time $\tau _p$ is prolonged such that $\tau _p>\tau _m$.

## Results & discussion

We study the effects of multi-exponential memory on the MSD in the absence of potentials, in the presence of harmonic confinement without energy barriers, and in double-well potentials with energy barriers. The comparison of the MSD in the presence and absence of memory effects in different potential landscapes allows us to determine the individual influence of memory, confinement, and energy barriers on the dynamics.

### Free diffusion

In SI Sec. II, we derive the analytic result for the MSD for a general multi-exponential kernel Eq. (4) in the presence of a harmonic potential $U(x)=Kx^2/2$ as

10 $$\begin{aligned}&C_\mathrm{{MSD}}(t) \nonumber \\&=\frac{B}{c_{n+2}} \left( \sum _{i=1}^{n+2}\frac{e^{-\sqrt{-\omega ^2_i} t} - 1}{\sqrt{-\omega ^2_i} \prod _{j\ne i} (\omega ^2_i-\omega ^2_j) } \sum _{m=1}^{2n-1}k_m \omega _i^{2m-2} \right) \,, \end{aligned}$$

where $c_i$, $k_i$, and $\omega _i$ are constants determined by the kernel parameters $\gamma _i$ and $\tau _i$, and by the harmonic coupling strength *K*. In the SI Sec. II, we give explicit examples of Eq. (10) for $n=3$ and $n=5$. To understand the impact of friction memory $\Gamma (t)$ on the MSD, it is instructive to first analyze GLE dynamics in the absence of an external potential, i.e., $U(x)=0$.

**Fig. 2:**
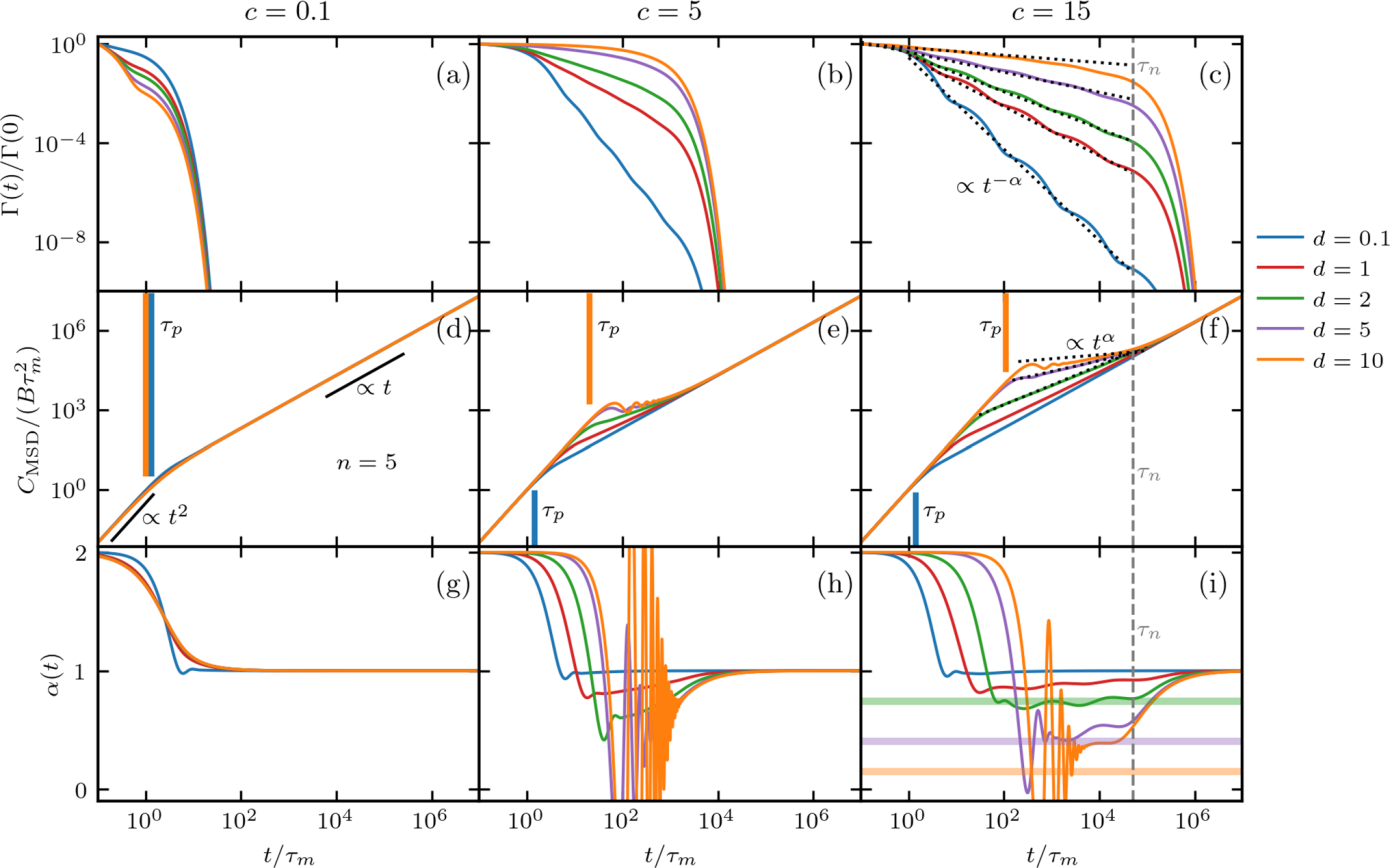
Non-Markovian dynamics in the absence of a potential ($U(x)=0$). Results are shown for $n=5$ and $\tau _1=\tau _m$. **a**–**c** Multi-exponential memory kernel $\Gamma (t)$ (Eq. (4)), for a range of *c* and *d* values, as defined by Eq. (5). The dotted lines in (**c**) represent the power law predicted by Eq. (6). **d**–**f** Analytic results for the MSD given by Eq. (10), determined by the friction kernels given in (**a**)–(**c**). The colored vertical lines show the persistence time $\tau _p$ for $d=0.1$ and $d=10$, defined in Eq. (11). The black lines in (**d**) indicate ballistic ($\alpha =2$) and diffusive ($\alpha =1$) scaling behavior. The dotted black lines in (**f**) show the predicted subdiffusive scaling of the MSDs for the dotted-line kernels in (**c**) for $c>d>1$, with the predicted $\alpha$ given by Eq. (6). **g**–**i** Time-dependent exponent $\alpha (t)$ of the MSDs in (**d**)-(**f**), obtained via Eq. (1). The horizontal lines in (**i**) represent the prediction of $\alpha$ by Eq. (6), where the color scheme matches the curves for $\alpha (t)$. The vertical dashed lines in (**c**), (**f**), and (**i**) show the longest memory time $\tau _n$

In Fig. 2, we present analytical results for MSDs Eq. (10) for $U(x) = 0$, which means $K=0$. The input memory kernels $\Gamma (t)$ are shown in Fig. 2a-c, the corresponding MSDs in Fig. 2d-f, and the time-dependent exponents $\alpha (t)$ in Fig. 2g-i. In Fig. 2a-c, we demonstrate that for certain combinations of *c* and *d*, the multi-exponential friction kernels exhibit extended regions following a power law $\Gamma (t)\propto t^{-\alpha }$, where $\alpha$ is predicted by Eq. (6) for $c > d$.

This is evident in Fig. 2c, where we plot the corresponding power laws using exponents from Eq. (6) as dotted lines. In Fig. 2f, we see that the region of the MSD between the short-time ballistic regime and the long-time diffusive regime is also described well by a power law $C_\mathrm{{MSD}}\propto t^\alpha$ [39, 54], with the same $\alpha$ that describes the memory kernels, when $\alpha <1$. The predicted exponents (Eq. (6)) agree well with $\alpha (t)$ (Eq. 1) in those regions, as validated by the horizontal lines in Fig. 2i. Beyond the longest memory time $\tau _n=\tau _1 c^{n-1}$, the kernels deviate from power-law behavior, clearly displaying an exponential decay for $t>\tau _n$. This corresponds to the crossover to the long-time diffusive regime with $\alpha =1$, as can be seen in Fig. 2c,f,i.

In Fig. 2d and g, where $c=0.1$, we observe the direct transition from the ballistic regime ($\alpha =2$) to long-time free diffusion ($\alpha =1$) at the inertial time $t=\tau _m$, as expected for $c<1$ and $d>1$ ($\tau _i<\tau _m$), which represents the Markovian limit. As demonstrated in Fig. 2, the ballistic regime extends with increasing *c* and *d*. The persistence time is given by the solution to the self-consistent equation

11 $$\begin{aligned} \tau _p = 1 / G(\tau _p)\,, \end{aligned}$$

shown as vertical lines in Fig. 2d-f and determines the end point of the ballistic regime in the MSD. The persistence time is the generalization of the inertial time in the case of time-dependent friction $\Gamma (t)$. In the absence of memory effects, $\Gamma (t)=2\delta (t)/\tau _m$ and *G*(*t*) instantaneously reaches $G(\infty )$. For short memory times ($\tau _i \ll \tau _m$), *G*(*t*) plateaus to $G(\infty )$ at $t<\tau _m$, such that $\tau _p=\tau _m$. However, for long memory times ($\tau _i\ge \tau _m$) the accumulation of friction is slow and less dissipation occurs at short times, resulting in an increased persistence time, $\tau _p$. As such, $\tau _p>\tau _m$ indicates the presence of memory effects with memory contributions satisfying $\tau _i\ge \tau _m$. All memory effects that contribute to parts of the total friction accumulating on times $t>\tau _m$, such as large values of *c* and *d*, prolong the ballistic regime in the MSD, as seen in Fig. 2d-f.

For certain combinations of *c* and *d*, we see that oscillations appear in the MSD. The amplitude and duration of these oscillations increase with increasing *d*/*c* when $c>1$, as can be seen when comparing Fig. 2h,i. When $c < 1$ and $d > 1$, or $c > 1$ and $d < 1$, and the first memory time is of the same order as the inertial time $\tau _m$, the form of Eq. (5) causes the kernel $\Gamma (t)$ to decay on the scale of $\tau _m$. As a result, the kernel integral *G*(*t*) saturates at $t \approx \tau _m$. In this case, a direct transition from the ballistic to the diffusive regime is observed in the MSD, which resembles the memoryless case, as in Fig. 2d. For increasing *c*, we also observe an increasingly long intermediate subdiffusive regime in the MSD up to the longest memory time $\tau _n=\tau _1 c^{n-1}$, as shown in Fig. 2a-c for $\tau _1=\tau _m$. The time-dependent exponent $\alpha (t)$ in Fig. 2i slightly oscillates, reflecting the individual exponential memory components, while its mean for $\tau _1<t<\tau _n$ is still given by Eq. (6). In the SI Sec. III, we show that for particularly large values of *c*, the minima in $\alpha (t)$ separate and additional intermediate diffusive regimes with $\alpha =1$ emerge.

**Fig. 3:**
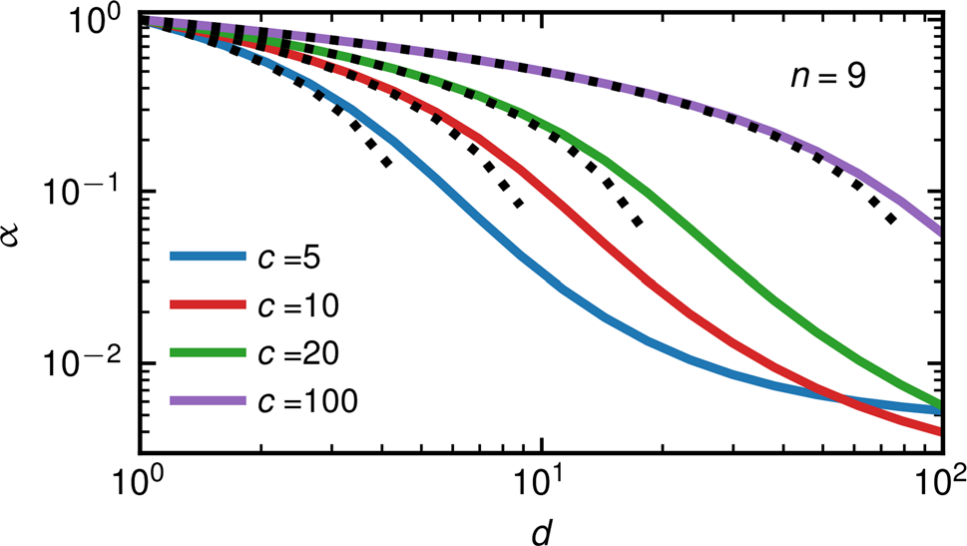
Exponent $\alpha$, obtained by fitting $\Gamma (t)\propto t^{-\alpha }$ to the multi-exponential kernel Eq. (4) with $n=9$ (solid lines), is compared to the prediction of Eq. (6) (dotted lines) for different *c* and *d*

In Fig. 3, we compare the prediction of Eq. (6) (dotted lines) to the scaling exponents $\alpha$ obtained by fitting power laws $\propto t^{-\alpha }$ to multi-exponential kernels $\Gamma (t)$ Eq. (4) (solid lines) over a finite range of time. The fitting details and examples for different values of *n* are given in SI Sec. III. For different *c* and *d*, Eq. (6) is seen to be a good description of the subdiffusive scaling exponent, as long as $c>d$, a condition required in the derivation of Eq. (6). This scaling of the friction kernel translates into the intermediate subdiffusive scaling of the MSD for $\tau _p<t<\tau _n$, provided that $\alpha <1$ [54], as shown in Fig. 2c,f. The certainty of an estimation of $\alpha$ from an experimental trajectory of finite length depends on the trajectory length. Thus, it is useful to estimate the error of experimentally obtained $\alpha$ values with Eq. (6). Such error estimates can be obtained by analytical considerations [55] or numerical methods [35].

### Diffusion in a harmonic potential

**Fig. 4:**
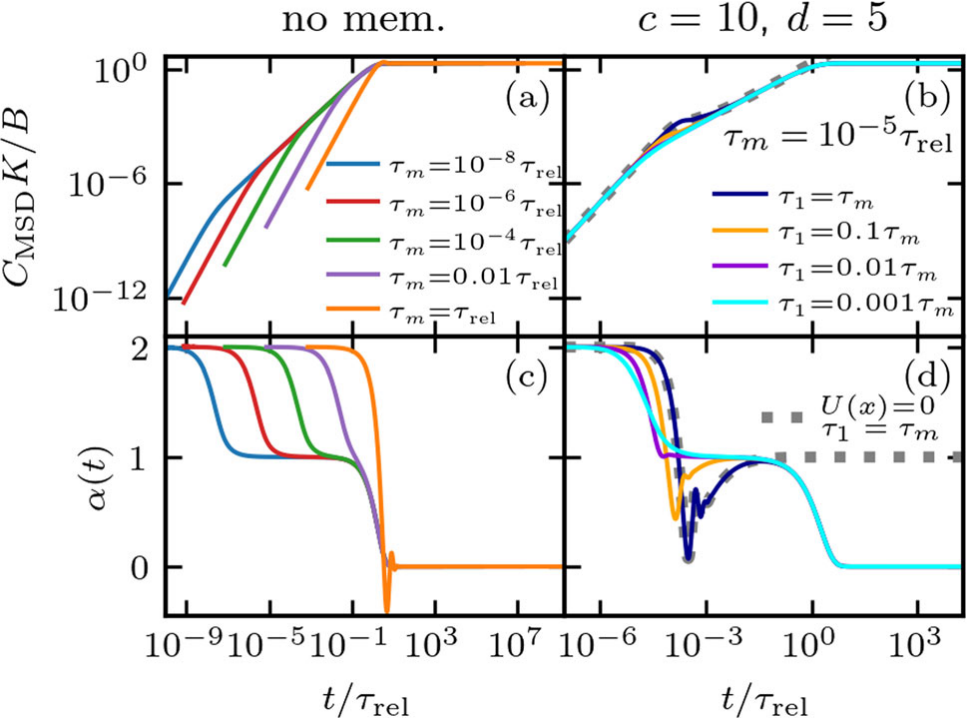
Analytical results for the MSD in harmonic confinement. The upper row depicts MSDs and the lower row the respective time-dependent exponent $\alpha (t)$ for (**a**),(**c**) without memory, governed by Eq. (9), and (**b**),(**d**) with $n=3$ exponential memory components, described by Eq. (4). The gray dotted lines in (**b**) and (**d**) represent the result in the absence of a potential ($K=0$) with the other parameters equivalent to the dark-blue line

Unlike the MSDs for freely diffusing observables, which tend to pure diffusion ($\alpha =1$) in the long-time limit, the MSD for motion in a confining potential tends to a constant plateau value given by $C_\mathrm{{MSD}}(\infty )=2\langle x^2 \rangle$. Indeed, our analytic result for the MSD in Eq. (10) for diffusion in a harmonic potential $U(x)=Kx^2/2$ (Fig. 4a and b) reach a finite plateau value with corresponding $\alpha (t)$ that tend to zero in the long-time limit, as shown in Fig. 4c and d.

In Fig. 4a, we see that the MSD for the memoryless case, described by Eq. (9), transitions from ballistic to diffusive behavior before reaching the long-time plateau. This occurs if the inertial time $\tau _m$ is small compared to the relaxation time in the harmonic potential given by

12 $$\begin{aligned} \tau _\mathrm{{rel}} = 1/(\tau _m K)\,. \end{aligned}$$

Eq. (12) defines the damping time of a harmonic oscillator with oscillation frequency $K^{1/2}$. It is the time scale at which the confinement effects due to the potential become important for the MSD. For systems with a persistence time shorter than the theoretical oscillation period, $\tau _p<K^{-1/2}$, no oscillations arise from the presence of the potential. In this case, all features in the MSD that occur on timescales shorter than $\tau _\mathrm{{rel}}$ are not influenced by the potential. In the memoryless limit, where $\tau _p = \tau _m$, oscillations in the MSD—and consequently in $\alpha (t)$—due to interactions with the potential occur only when $\tau _m$ is close to or larger than $\tau _\mathrm{{rel}}$. Such oscillations can be seen in Fig. 4c for the orange line, where $\tau _m=\tau _\mathrm{{rel}}=K^{-1/2}$. The motion in a harmonic potential with memory exhibits the same features in the MSD as for the free case on time scales smaller than the relaxation time $\tau _\mathrm{{rel}}$, as seen by comparing the dark-blue line with the gray dotted line in Fig. 4b,d. Furthermore, the data in Fig. 4b and d exhibit the limit of the standard Markovian Langevin dynamics when all memory times are shorter than the inertial time, $\tau _i\ll \tau _m$, leading to a direct transition from $\alpha =2$ to $\alpha =1$ at $t=\tau _m$ for the cyan line.

### Memoryless diffusion in the presence of an energy barrier

**Fig. 5:**
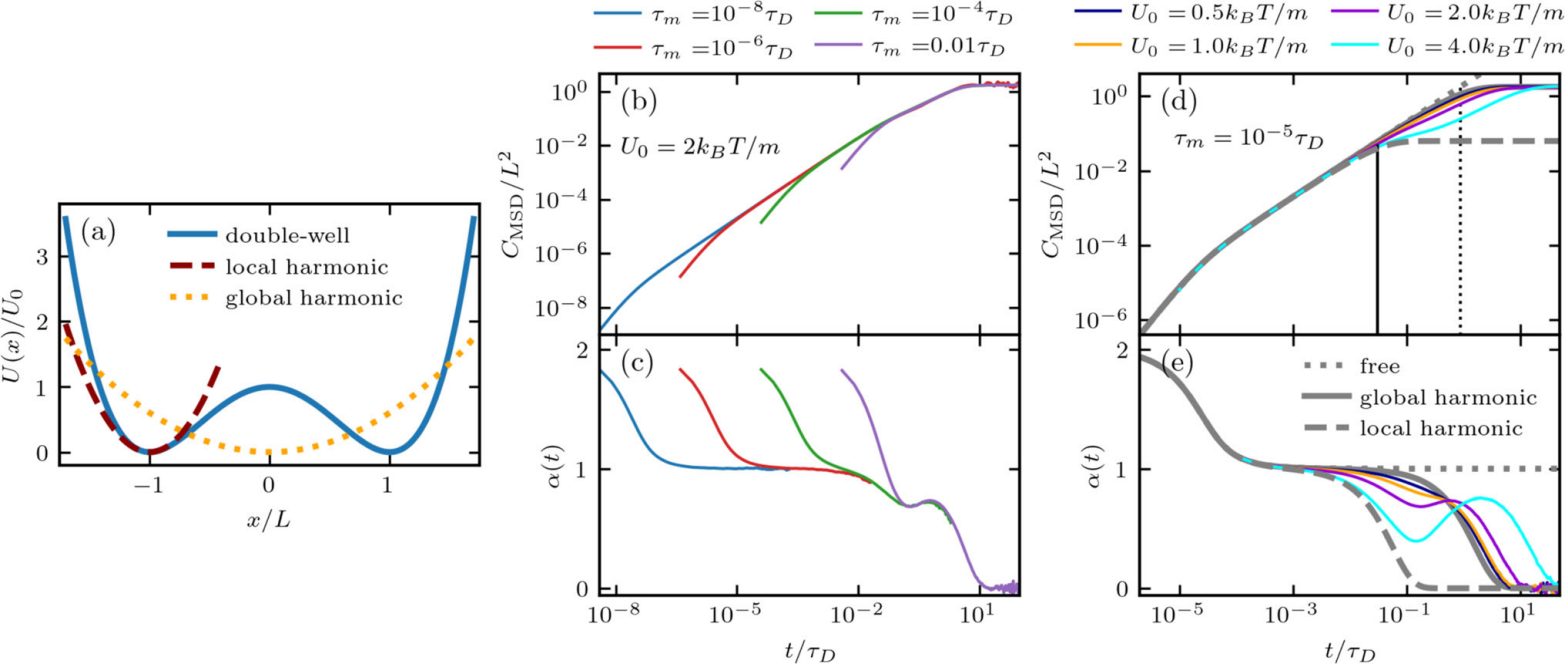
**a** Double-well potential (Eq. (13), blue solid line) with corresponding local harmonic approximation around $x/L=-1$ (brown dashed line). The global harmonic potential (orange dotted line) leads to the same positional variance $\langle x^2\rangle$ as the double-well potential. **b**–**e** MSDs and time-dependent exponents $\alpha (t)$ for simulations in a double-well potential without memory; **b** and **c** results for different inertial times $\tau _m$ with fixed barrier height $U_0=2k_BT/m$. **d** and **e** Results for different barrier heights $U_0$ with fixed $\tau _m=10^{-5}\tau _D$. The dotted gray curves in (**d**) and (**e**) represent the analytical result for $U(x)=0$ with the same memory kernel parameters as used in the simulations. The solid gray curves represent the analytical result in the global harmonic potential, and the dashed gray curves represent the analytical result in the local harmonic potential, both for $U_0=4B$. The solid vertical line in (**d**) shows $\tau _\mathrm{{rel}}^\mathrm{{loc}}$ (Eq. (14)), and the vertical dotted line shows $\tau ^\mathrm{{glob}}_\mathrm{{rel}}$ (see text), both for $U_0=4B$

Systems with distinct meta-stable states, e.g., proteins, chemical reactants, or particles moving across coexisting phases, exhibit energy barriers, which can lead to subdiffusion [51, 56]. However, as we demonstrated in the previous sections, memory-dependent friction by itself can lead to subdiffusion, even in the absence of a potential or energy barrier. To distinguish barrier from memory effects, we introduce the double-well potential

13 $$\begin{aligned} U(x) = U_0 \left( \left( \frac{x}{L}\right) ^2 - 1 \right) ^2\,, \end{aligned}$$

with barrier height $U_0$, which is a simplified, symmetric analog of the potential for the $\alpha _3$D protein shown in Fig. 1a. Since it is analytically intractable to derive the MSD for the dynamics of a GLE in a non-harmonic potential, we turn to simulations [57]. Here, we introduce the diffusion time $\tau _D=L^2/(B\tau _m)$, which is the average time to diffuse over a characteristic length *L* in the absence of a potential. In the case of the double-well potential, we set *L* as the distance between the minimum and the barrier position, as shown in Fig. 5a.

In Fig. 5b-e, we discuss the effect of energy barriers on the MSD for a memoryless system. In Fig. 5b and c, we show that for small inertial times $\tau _m \ll \tau _D$, there are extended regions of free diffusion, characterized by $\alpha =1$, in the MSD. That is, given that $\tau _p<K_\mathrm{{loc}}^{-1/2}$, which assures the absence of harmonic oscillations that might otherwise arise due to the presence of the potential. For times greater than the local harmonic relaxation time, defined by

14 $$\begin{aligned} \tau _\mathrm{{rel}}^\mathrm{{loc}} = 1/(\tau _m K_\mathrm{{loc}})\,, \end{aligned}$$

the observable is influenced by the local potential well, where $K_\mathrm{{loc}}$ is the single-well harmonic strength, given by $K_\mathrm{{loc}}=8U_0/L^2$ (Fig. 5a). The system behaves much like the harmonically confined system discussed in the previous section, as seen by comparing Fig. 4a,c to Fig. 5b,c. However, in the case of the double-well potential, we observe the onset of additional subdiffusive behavior for $t>\tau _\mathrm{{rel}}^\mathrm{{loc}}$, characterized by non-monotonic behavior of $\alpha (t)$ (Fig. 5c), which, as discussed previously [51, 56], is entirely due to the barrier-crossing dynamics. For barrier heights $U_0<2B$, the MSD is well captured by the analytical result in a harmonic potential with a coupling strength $K_\mathrm{{glob}}$, chosen such that $B/K_\mathrm{{glob}}=\langle x^2 \rangle$, where we compute the variance, $\langle x^2 \rangle \propto \int _{-\infty }^{\infty } x^2 e^{-U(x)/B} dx$, numerically (Fig. 5d,e). $K_\mathrm{{glob}}$ is the effective global harmonic coupling strength associated with the quartic double-well potential. As such, $\tau _\mathrm{{rel}}^\mathrm{{glob}}$ is the global relaxation time obtained by using $K=K_\mathrm{{glob}}$ in Eq. (12). The agreement between the harmonic prediction with $K_\mathrm{{glob}}$ and the MSD extracted from the double-well simulations for sufficiently low barriers ($U_0<2B$) indicates that the main effect of the double-well potential is the confinement at long times. In fact, for such small barriers, we find that the simulation MSDs agree well with the analytical result in the absence of an external potential up to the relaxation time of the global harmonic potential $\tau _\mathrm{{rel}}^\mathrm{{glob}}$ (vertical dotted line in Fig. 5d). This further emphasizes that the MSD is insensitive to sufficiently low barriers. It should be noted that such low barriers are observed in fast-folding proteins [42] or small polypeptides [41]. However, these systems are known to be strongly non-Markovian. As we will see in the next section, memory has no influence on this insensitivity to low barriers.

As we see in Fig. 5a, the double-well potential can be approximated by a harmonic potential around a minimum. Therefore, we can describe the short-time dynamics by our analytical results in a harmonic potential, which is suitable for times less than the relaxation time in the local well $\tau _\mathrm{{rel}}^\mathrm{{loc}}$, which we show as a vertical solid black line in Fig. 5d for $U_0=4B$. From the simulation results in Fig. 5d,e, we see that, for short times ($t<\tau _\mathrm{{rel}}^\mathrm{{loc}}$), the MSD in a double-well potential does indeed follow the prediction of the local harmonic well. The dip in the time-dependent exponent $\alpha (t)$ for $t>\tau _\mathrm{{rel}}^\mathrm{{loc}}$, observed in Fig. 5e, becomes more pronounced with increasing barrier height. It originates from the combined effect of local confinement at short times followed by barrier crossing. Therefore, potential energy landscapes with sufficiently high energy barriers can induce subdiffusive behavior in the MSD, even in the absence of memory effects. In the case of multiple energy barriers and for weakly localizing potentials, barrier-induced subdiffusion can persist up to long times, however, the asymptotic long-time MSD is dominated by the memory for sufficiently long memory contributions [58, 59].

### Diffusion in the presence of memory and energy barriers

**Fig. 6:**
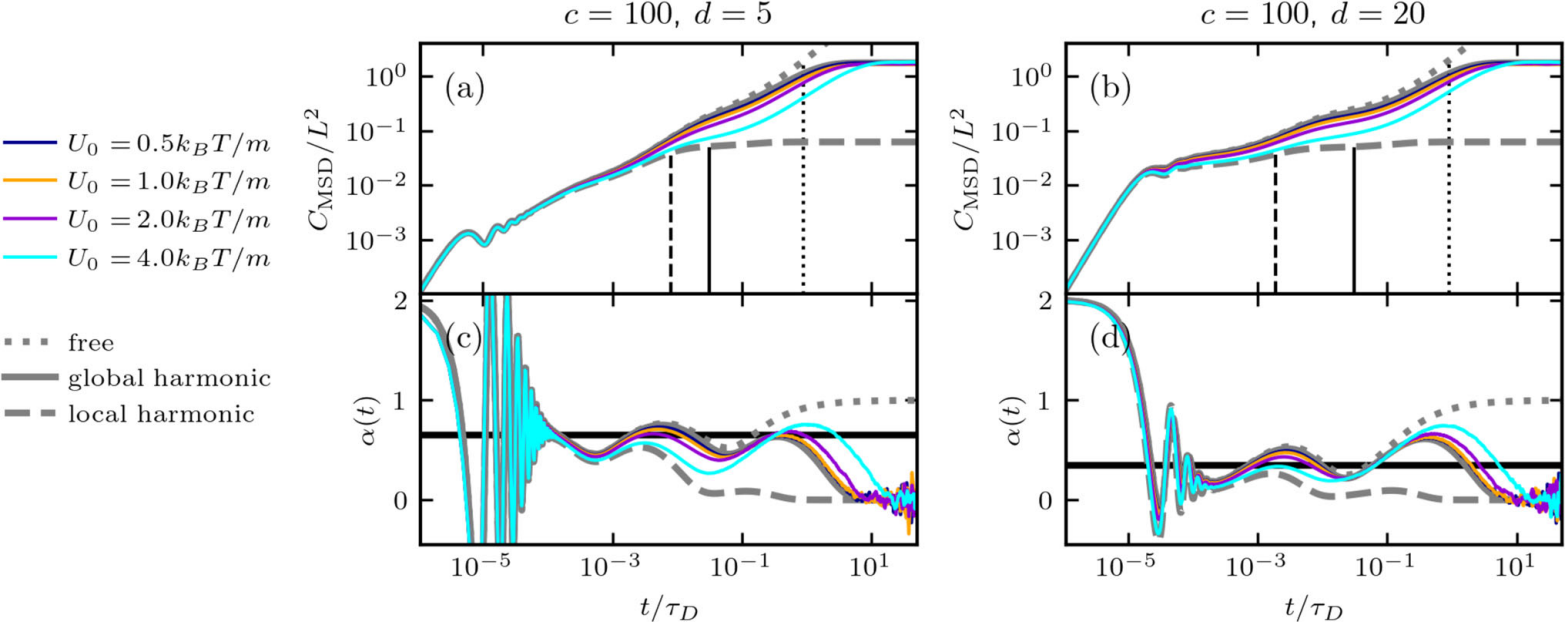
MSDs and time-dependent exponents $\alpha (t)$ from GLE simulations in a double-well potential with multi-exponential memory ($n=3$) over a range of potential energy barrier heights $U_0$. The inertial time is $\tau _m=10^{-8}\tau _D$, and the shortest memory time is $\tau _1=10^3\tau _m$. **a** and **c** Memory time ratio $c=100$ and friction-amplitude ratio $d=5$, as defined in Eq. (5). **b** and **d** $c=100$ and $d=20$. The dotted gray curves represent the analytical result for $U(x)=0$ with the same memory kernel parameters as used in the simulations. The solid gray curves represent the analytical result in the global harmonic potential, and the dashed gray curves represent the analytical result in the local harmonic potential, both for $U_0=4B$. The solid vertical lines show $\tau _\mathrm{{rel}}^\mathrm{{loc}}$ (Eq. (14)), the dashed vertical lines show $\tau _\mathrm{{rel}}^\mathrm{{loc*}}$ (Eq. (15)), and the dotted vertical lines show $\tau ^\mathrm{{glob*}}_\mathrm{{rel}}$ (Eq. (16)), all for $U_0=4B$

We now compare subdiffusion that is generated by memory effects to barrier-induced subdiffusion in a double-well potential. Simulations in the presence of memory are performed using Markovian embedding techniques [57, 60–62], simulation details are given in SI Sec. IV. In Fig. 6a and b, we show MSDs for systems with memory kernels characterized by two different combinations of *c* and *d*, as defined by Eq. 5. We introduce a local harmonic relaxation time for systems with memory, $\tau _\mathrm{{rel}}^\mathrm{{loc*}}$, which is the timescale at which the friction force is balanced by the local harmonic restoration force, given by the solution to the self-consistent equation

15 $$\begin{aligned} \tau _\mathrm{{rel}}^\mathrm{{loc*}} = G(\tau _\mathrm{{rel}}^\mathrm{{loc*}}) / K_\mathrm{{loc}}\,, \end{aligned}$$

which is determined numerically using an iteration procedure, similar to the persistence time $\tau _p$ defined in Eq. 11. This prediction of $\tau _\mathrm{{rel}}^\mathrm{{loc*}}$, shown as dashed vertical lines for $U_0=4B$ in Fig. 6a and b, agrees with the time at which the MSD of the double-well simulation deviates from the analytical result in the local harmonic well (dashed gray line). $\tau _\mathrm{{rel}}^\mathrm{{loc*}}$ is smaller than the corresponding $\tau _\mathrm{{rel}}^\mathrm{{loc}}$, because the latter incorporates the total friction integral $G(\infty )$, while the former depends on an incomplete accumulation of friction, since $\Gamma (t)$ acts over a range of time scales. When friction is dominated by contributions with long memory times, which can be achieved by increasing *d* for fixed *c* (see Fig. 2), faster processes experience less friction. This has the effect of reducing $\tau _\mathrm{{rel}}^\mathrm{{loc*}}$, which can be seen by comparing the positions of the vertical dashed lines in Fig. 6a and b.

Similarly, we introduce the relaxation time in the global potential for systems with memory-dependent friction, $\tau ^\mathrm{{glob*}}_\mathrm{{rel}}$, which marks the deviation between the freely diffusing MSD and the MSD in the global harmonic well and is defined by

16 $$\begin{aligned} \tau ^\mathrm{{glob*}}_\mathrm{{rel}} = G(\tau ^\mathrm{{glob*}}_\mathrm{{rel}}) / K_\mathrm{{glob}}\,. \end{aligned}$$

Equation 16 is analogous to Eq. (12) for systems with memory. In Fig. 6a and b, we see that $\tau ^\mathrm{{glob*}}_\mathrm{{rel}}$, indicated with dotted vertical lines, aligns precisely with the deviation times between the free diffusion MSD and the MSDs in the global harmonic potential. Much like in the case of local relaxation, where $\tau _\mathrm{{rel}}^\mathrm{{loc*}} <\tau _\mathrm{{rel}}^\mathrm{{loc}}$, the analog for the global relaxation is $\tau _\mathrm{{rel}}^\mathrm{{glob*}}<\tau _\mathrm{{rel}}^\mathrm{{glob}}$, i.e., the relaxation times in the presence of memory are always smaller than the relaxation times in the corresponding Markovian limits. These conditions are satisfied for any monotonically decreasing friction kernel. For low barriers (up to heights $U_0=2B$), we see in Fig. 6c and d that the time-dependent exponent $\alpha (t)$ is well described by the analytical free-diffusion prediction (dotted lines) for $t<\tau ^\mathrm{{glob*}}_\mathrm{{rel}}$. This means that for low enough barriers, the dynamics are dominated by the friction memory for $t<\tau ^\mathrm{{glob*}}_\mathrm{{rel}}$, and that the energy barriers have negligible influence on the system dynamics. In general, the MSD and the corresponding $\alpha (t)$ in Fig. 6 for a particular memory kernel $\Gamma (t)$ are always bounded between the limiting free-diffusion behavior and the local harmonic approximation. The shape of the local potential becomes increasingly influential for large barrier heights. We observe that the subdiffusive contribution to the MSDs for $t<\tau _\mathrm{{rel}}^\mathrm{{loc*}}$ exhibits an exponent $\alpha$ close to the prediction by Eq. (6) (solid horizontal black line in Fig. 6c and d), regardless of barrier height. Therefore, even for systems with large barriers, memory effects still contribute significantly to the overall subdiffusivity.

Taken together, we can summarize the results in Figs. 5 and 6 as follows: For small inertial times ($\tau _m\ll \tau _D$) and low energy barriers ($U_0 < 2B$), the dynamics are dominated by memory effects until $t=\tau ^\mathrm{{glob*}}_\mathrm{{rel}}$, after which the global confinement dominates. For higher barrier heights, the dynamics exhibit memory-induced subdiffusivity until $t=\tau _\mathrm{{rel}}^\mathrm{{loc*}}$, after which the subdiffusive dynamics are due to a combination of memory effects and the shape of the potential. Clearly, the presence of energy barriers leads to additional subdiffusive behavior, in line with previous studies [51, 56]. However, our findings suggest that, especially for small barrier heights and highly damped systems, the dynamics are dominated by memory effects, in line with recent findings for protein folding [42, 43].

## Conclusions

We analyze the dynamics of an observable experiencing multi-exponential friction memory in the framework of the generalized Langevin equation in the presence and absence of potential energy profiles *U*(*x*). We find that, in the absence of a potential, $U(x)=0$, exponential memory components with exponentially spaced memory times and amplitudes produce subdiffusive behavior with the characterizing MSD exponent $\alpha$ predicted by Eq. (6). The short-time ballistic regime of the MSD, characterized by $\alpha =2$, is prolonged by memory effects and its termination is captured by the persistence time $\tau _p$, which is a generalization of the inertial time $\tau _m$ for systems with memory and defined in Eq. (11). In the presence of energy barrier heights $U_0\lesssim 2B$ (corresponding to $U_0\lesssim 2k_BT/m$), dynamics are dominated by memory effects for times smaller than the global harmonic relaxation time $t<\tau ^\mathrm{{glob*}}_\mathrm{{rel}}$, defined in Eq. (16), and are well described by analytical results in an appropriately chosen global harmonic potential. In particular, the subdiffusive scaling $\alpha$ is predicted by the same Eq. (6) as in the case without external potential, highlighting the importance of memory effects compared to effects due to the potential. In the case of strong damping $\tau _m\ll \tau _D$, the global harmonic relaxation time $\tau ^\mathrm{{glob*}}_\mathrm{{rel}}$ becomes large such that the dynamics are governed by memory effects for long times.

Effects of the potential landscape on the MSD become important for barrier heights above $U_0\sim 2B$. Similarly to previous findings [56, 63], we observe a subdiffusive regime for sufficiently high energy barriers. Nevertheless, the dynamics in local minima are well described by our analytical results up to the local relaxation time $\tau _\mathrm{{rel}}^\mathrm{{loc*}}$, defined in Eq. (15), which depends on both the local shape of the potential and the memory, as demonstrated by simulations in a double-well potential. For shorter times $t<\tau _\mathrm{{rel}}^\mathrm{{loc*}}$, memory effects dominate the dynamics and after that, memory effects still influence the MSD significantly.

Overall, we demonstrate that in order to accurately describe dynamics in subdiffusive systems, one must correctly account for both the potential energy landscape and memory effects. By disentangling contributions to subdiffusion by the potential landscape and memory effects, we confirm that, in many instances, energy barriers are less influential than friction in determining the system dynamics, as was shown recently to be the case for fast-folding proteins [42]. However, our analysis is general and our findings are expected to have implications beyond the example of protein folding, shedding light on the origin of subdiffusive dynamics in a wide range of physical, chemical, and biological systems.

## Supplementary Information

Below is the link to the electronic supplementary material.

Supplementary file 1 (pdf 1018 KB)

## Data Availability

The code to simulate the GLE and implementations of the MSD formulas for multi-exponential memory are available at https://github.com/kanton42/msd_subdiffusion
