# Supplementary material for: Subdiffusion from competition between multi-exponential friction memory and energy barriers
Source: Eur Phys J E Soft Matter. 2025 Sep 12;48(8-9):55. doi: 10.1140/epje/s10189-025-00518-y (PMC12431924; doi:10.1140/epje/s10189-025-00518-y)
Supplement: Supplementary file 1 — (pdf 1018 KB) [file 10189_2025_518_MOESM1_ESM.pdf]

Supplementary information: Subdiffusion from competition between multi-exponential friction memory and energy barriers

Anton Klimek[1], Benjamin A. Dalton[1], Roland R. Netz[1*]

[1]Fachbereich Physik, Freie Universität Berlin, Arnimalle 14, Berlin, 14195, Germany.

I Determining MSD exponent α from multiexponential memory

Starting from the friction kernel expression given in the main text Eq. (4) with memory-time scaling c and friction-coefficient scaling d according to Eq. (5), one obtains the values of the kernel $\Gamma(t_i) = \Gamma_i$ at times $t_i = \tau_1 c^{i-1}$ approximately as

$$\Gamma_i = e^{-1}\frac{\gamma_1}{\tau_1}\sum_{j=i}^{n}\frac{d^{j-1}}{c^{j-1}}\,, \tag{S1}$$

where contributions $j < i$ are neglected, as they are exponentially small for $c > 1$.We define the ratio of the scaling factors $b = d/c$ and split the sum in Eq. (S1), which leads to

$$\Gamma_i = e^{-1}\frac{\gamma_1}{\tau_1}\left(\sum_{j=i}^{\infty} b^{j-1} - \sum_{j=n+1}^{\infty} b^{j-1}\right) \tag{S2}$$

$$= e^{-1}\frac{\gamma_1}{\tau_1}\left(b^{i-1}\sum_{k=0}^{\infty} b^{k} - b^{n}\sum_{k=0}^{\infty} b^{k}\right)\,. \tag{S3}$$

In the case of $b < 1$, i.e. $c > d$, the geometric sum converges to $\sum_{k=0}^{\infty} b^k = 1/(1-b)$. Thus the kernel at time t_i is given by

$$\Gamma_i = e^{-1}\frac{\gamma_1}{\tau_1}\frac{b^{i-1}-b^{n}}{1-b}\,. \tag{S4}$$

The logarithm of Eq. (S4) reads

$$\ln(\Gamma_i) = 1 + \ln\left(\frac{\gamma_1}{\tau_1}\right) + (i-1)\ln(b) + \ln(1-b^{n-i+1}) - \ln(1-b)\,, \tag{S5}$$

which leads to the logarithmic derivative between t_i and t_{i+1} as

$$\frac{\mathrm{d}\ln(\Gamma(\mathrm{t}))}{\mathrm{d}\ln(\mathrm{t})}\Big|_{t=t_i} = \frac{\ln(\Gamma_{i+1}) - \ln(\Gamma_i)}{\ln(t_{i+1}) - \ln(t_i)} = \frac{\ln(b) + \ln(1-b^{n-i}) - \ln(1-b^{n-i+1})}{\ln(c)}\,. \tag{S6}$$

In the case of $n \gg i-1$, the logarithmic derivative Eq. (S6) simplifies to

$$\frac{\mathrm{d}\ln(\Gamma(\mathrm{t}))}{\mathrm{d}\ln(\mathrm{t})} = \frac{\ln(b)}{\ln(c)} = \frac{\ln(d/c)}{\ln(c)}\,. \tag{S7}$$

This logarithmic derivative is the exponent of the friction kernel $\Gamma(t) \propto t^{-\alpha}$, as shown in Fig. 2 in the main text. Since a scaling of the friction kernel $\Gamma(t) \propto t^{-\alpha}$ leads to $C_{\mathrm{MSD}}(t) \propto t^{\alpha}$, for $\alpha < 1$ [54], we can estimate

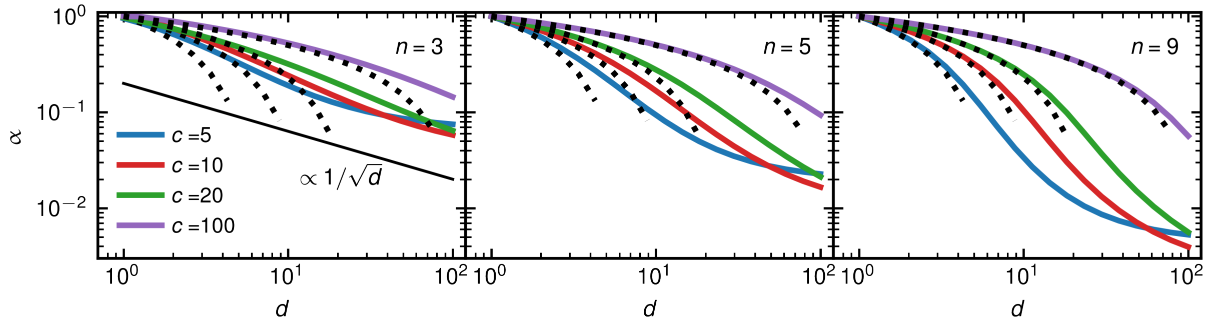

Fig. S1 The behavior of α obtained from fits of $\Gamma(t) \propto t^{-\alpha}$ to the multiexponential kernel Eq. (4) is compared to the prediction by Eq. (S8) (dotted lines) for different c and d and for $n = 3$, $n = 5$ and $n = 9$.

the scaling exponent of the MSD via Eq. (S7) as a function of c and d as

$$\alpha(c, d) = \frac{\ln(c/d)}{\ln(c)} \,. \tag{S8}$$

In Fig. S1 we compare the result of Eq. (S8) to the α values obtained from double-logarithmic fits of $\Gamma(t) \propto t^{-\alpha}$ to the friction kernel Eq. (4) between $\tau_1 < t < \tau_n$ for different values of c, d, and n. The fits minimize the squared difference of the logarithms on a logarithmically spaced time axis between $\tau_1 < t < \tau_n$ for $n = 9$ exponentials. As expected from the derivation, Eq. (S8) describes the scaling exponent α most accurately for high numbers of exponential kernel contributions n, shown by the good agreement between fitted α and Eq. (S8) for $n = 9$ in Fig. S1c. But even for $n = 3$ and $n = 5$ the scaling exponent of the friction kernel $\Gamma(t)$ is well predicted for $c > d$, where the deviation of the prediction Eq. (S8) to the observed scaling becomes larger with increasing d, as expected by the assumption $c > d$ in the derivation.

In contrast to the subdiffusive regimes originating from positive exponential friction components, it was shown before that negative exponential friction components lead to additional intermediate ballistic regimes [29].

In the long time limit, α always tends towards the long time diffusive value of $\alpha = 1$, and thus, the estimation of the scaling exponent by Eq. (S8) is not applicable for long times $t > \tau_n$. Clearly, this is expected since exponential memory kernels involve a longest time scale, in contrast to power-law memory kernels, which lead to subdiffusive behavior on all time scales when not using short-time and long-time cutoffs. Examples of the transition to the long-time diffusive behavior at $t = \tau_n$ are shown in Figs. S2d,h.

II Derivation of MSD for the GLE with multi-exponential memory

In Fourier space, the GLE (Eq. (2)) can be written as

$$\tilde{x}(\omega) = \tilde{\chi}(\omega)\tilde{F}_R(\omega) \,, \tag{S9}$$

where $\tilde{\chi}(\omega)$ is the Fourier transform of the position response function, which for a harmonic potential $U(x) = Kx^2/2$ takes the form

$$\tilde{\chi}(\omega) = \left(K - \omega^2 + i\omega\tilde{\Gamma}_+(\omega)\right)^{-1} \tag{S10}$$

with $\tilde{\Gamma}_+(\omega)$ being the half-sided Fourier transform of the memory kernel, as defined via

$$\tilde{\Gamma}_+(\omega) = \int_0^\infty e^{-i\omega t}\Gamma(t)dt \,. \tag{S11}$$

The Fourier transform of the position correlation function $C_{xx}(t) = \langle x(0)x(t)\rangle$ can be written as

$$\tilde{C}_{xx}(\omega) = B\tilde{\chi}(\omega)\tilde{\Gamma}(\omega)\tilde{\chi}(-\omega) \,, \tag{S12}$$

where we made use of the relation between the second moment of the random force and $\Gamma(t)$, Eq. (3), and the mean squared velocity $B = \langle \dot{x}^2 \rangle$. Equation (S12) can be rewritten as

$$\tilde{C}_{xx}(\omega) = -\frac{B}{i\omega}\left(\tilde{\chi}(\omega) - \tilde{\chi}(-\omega)\right) , \tag{S13}$$

which leads to the MSD given as

$$\begin{aligned} C_{\mathrm{MSD}}(t) &= 2(C_{xx}(0) - C_{xx}(t)) \\ &= B\int_{-\infty}^{\infty} \frac{d\omega}{\pi}\frac{e^{i\omega t}-1}{i\omega}\left(\tilde{\chi}(\omega) - \tilde{\chi}(-\omega)\right) . \end{aligned} \tag{S14}$$

Generally, the response function for a sum of n exponentials kernel

$$\Gamma(t) = \sum_i^n a_i e^{-t/\tau_i} \tag{S15}$$

can be written for a harmonic potential as

$$\tilde{\chi}(\omega) = \left(K - \omega^2 + \sum_{j=1}^{n} \frac{i\omega a_j}{i\omega + \frac{1}{\tau_j}}\right)^{-1} , \tag{S16}$$

where we defined $a_i = \frac{\gamma_i}{\tau_i}$. Inserting Eq. (S16) into the MSD integral, Eq. (S14), one obtains

$$C_{\mathrm{MSD}}(t) = B\int_{-\infty}^{\infty}\frac{d\omega}{\pi}\frac{-2p_1(e^{i\omega t}-1)\sum_j^n \frac{a_j}{\tau_j}p_2(m\neq j)}{p_1p_2(K^2-2K\omega^2+\omega^4)+2\omega^2(K-\omega^2)p_1\sum_j^n a_j p_2(j\neq m)+p_2\sum_{j,k}^n \omega^2 a_j a_k p_1(l,m\neq j,k)} , \tag{S17}$$

with

$$p_1 = \prod_{l,m}^{n}\left(\omega^2 + \frac{i\omega}{\tau_m} - \frac{i\omega}{\tau_l} + \frac{1}{\tau_l\tau_m}\right) \tag{S18}$$

and

$$p_2 = \prod_{m=1}^{n}\left(\omega^2 + \frac{1}{\tau_m^2}\right) . \tag{S19}$$

$p_1(l, m \neq j, k)$ denotes p_1 leaving out the indices $l = j$ and $m = k$ in the multiplication Eq. (S18) and similarly, $p_2(m \neq j)$ denotes p_2 without the inclusion of the term with $m = j$ in Eq. (S19). From Eq. (S17), one can bring the MSD into the form

$$C_{\mathrm{MSD}}(t) = B\int_{-\infty}^{\infty}\frac{d\omega}{\pi}\frac{\sum_i^{2n-1} k_i\omega^{2(i-1)}}{c_{n+2}\prod_{i=0}^{n+2}(\omega^2-\omega_i^2)} \tag{S20}$$

with ω_i^2 being the solutions to the $n+2$ order polynomial in ω^2

$$\sum_{i=0}^{n+2} c_i\omega^{2i} = 0 . \tag{S21}$$

The constants c_i and k_i can be read off by comparing Eqs. (S20) and (S17), where c_i are not to be confused with the memory time ratio c. The MSD takes the form

$$C_{\mathrm{MSD}}(t) = \frac{B}{c_{n+2}}\left(\sum_{i=1}^{n+2}\frac{e^{-\sqrt{-\omega_i^2}t}-1}{\sqrt{-\omega_i^2}\prod_{j\neq i}(\omega_i^2-\omega_j^2)}\sum_{m=1}^{2n-1}k_m\omega_i^{2m-2}\right) , \tag{S22}$$

In the case $K = 0$, Eq. (S17) simplifies to

$$C_{\mathrm{MSD}}(t) = B\int_{-\infty}^{\infty}\frac{d\omega}{\pi}\frac{-2p_1(e^{i\omega t}-1)\sum_j^n \frac{a_j}{\tau_j}\prod_{j\neq m}(\omega^2+\frac{1}{\tau_m^2})}{\omega^4 p_1\left[p_2 - 2\sum_j^n a_j\prod_{j\neq m}(\omega^2+\frac{1}{\tau_m^2})\right] + p_2\sum_{j,k}^n \omega^2 a_j a_k \prod_{l,m\neq j,k}(\omega^2+\frac{i\omega}{\tau_l}-\frac{i\omega}{\tau_m}+\frac{1}{\tau_l\tau_m})}\,. \tag{S23}$$

and the MSD takes the form

$$C_{\mathrm{MSD}}(t) = B\int_{-\infty}^{\infty}\frac{d\omega}{\pi}\frac{\sum_i^{2n-1} k_i\omega^{2(i-1)}}{c_{n+2}\prod_{i=1}^{n+2}(\omega^2-\omega_i^2)}, \tag{S24}$$

with ω_i^2 now given by the roots of the $n+1$ order polynomial

$$\sum_{i=1}^{n+2} c_i\omega^{2i-2} = 0\,. \tag{S25}$$

The MSD for $K = 0$ thus always takes the form

$$C_{\mathrm{MSD}}(t) = \frac{B}{c_{n+2}}\left(\frac{(-1)^n k_1 t}{\prod_{j=1}^n \omega_j^2} + \sum_{i=1}^{n+1}\frac{e^{-\sqrt{-\omega_i^2}t}-1}{\sqrt{-\omega_i^2}\prod_{j\neq i}(\omega_i^2-\omega_j^2)}\sum_{m=1}^{2n-1}k_m\omega_i^{2m-4}\right)\,. \tag{S26}$$

Here, the first term proportional to t determines the long time diffusivity, since the other terms decay exponentially as a function of time. As an example, we now consider the case of $K = 0$ and $n = 3$ in the memory kernel Eq. (S15). From Eq. (S23) one obtains

$$C_{\mathrm{MSD}}(t) = B\int_{-\infty}^{\infty}\frac{d\omega}{\pi}\frac{(e^{i\omega t}-1)(k_1+k_2\omega^2+k_3\omega^4)}{\omega^2(c_1+c_2\omega^2+c_3\omega^4+c_4\omega^6+c_5\omega^8)}\,. \tag{S27}$$

where the constants c_i and k_i are given as

$$\begin{aligned}
k_1 =& -2(a_1\tau_1 + a_2\tau_2 + a_3\tau_3)\\
k_2 =& -2(a_2\tau_1^2\tau_2 + a_1\tau_1\tau_2^2 + a_3\tau_1^2\tau_3 + a_3\tau_2^2\tau_3 + a_1\tau_1\tau_3^2 + a_2\tau_2\tau_3^2)\\
k_3 =& -2\tau_1\tau_2\tau_3(a_3\tau_1\tau_2 + a_2\tau_1\tau_3 + a_1\tau_2\tau_3)\\
c_1 =&(a_1\tau_1 + a_2\tau_2 + a_3\tau_3)^2\\
c_2 =&1 - 2a_2\tau_2^2 + \tau_1^2(-2a_1 + (a_1+a_2)^2\tau_2^2) + 2a_3\tau_1\tau_2(a_2\tau_1 + a_1\tau_2)\tau_3 + ((a_1\tau_1 + a_2\tau_2)^2 + a_3^2(\tau_1^2+\tau_2^2)\\
&+2a_3(-1 + a_1\tau_1^2 + a_2\tau_2^2))\tau_3^2\\
c_3 =&\tau_3^2 + \tau_2^2(1 - 2(a_2+a_3)\tau_3^2) + \tau_1^2(1 - 2(a_1+a_2)\tau_2^2 + (-2(a_1+a_3) + (a_1+a_2+a_3)^2\tau_2^2)\tau_3^2)\\
c_4 =& -\tau_2^2\tau_3^2 + \tau_1^2(-\tau_3^2 + \tau_2^2(-1 + 2(a_1+a_2+a_3)\tau_3^2))\\
c_5 =&\tau_1^2\tau_2^2\tau_3^2
\end{aligned} \tag{S28}$$

Regarding the integral in Eq. (S27) as a sum of the three terms containing k_i, we see that all terms have the same poles, where the term containing k_1 has the additional double pole at $\omega = 0$. The solutions to

$$c_1 + c_2\omega^2 + c_3\omega^4 + c_4\omega^6 + c_5\omega^8 = 0\,, \tag{S29}$$

which we denote by ω_i^2, define the remaining poles of the integral Eq. (S27) as $\pm\sqrt{\omega_i^2}$. We rewrite the polynomial Eq. (S29) using the roots ω_i^2 as

$$c_1 + c_2\omega^2 + c_3\omega^4 + c_4\omega^6 + c_5\omega^8 = c_5(\omega^2 - \omega_1^2)(\omega^2 - \omega_2^2)(\omega^2 - \omega_3^2)(\omega^2 - \omega_4^2)\,. \tag{S30}$$

Next we use the partial fraction decompositions

$$\frac{1}{\omega^2 \prod_{i=1}^{k}(\omega^2 - \omega_i^2)} = \frac{(-1)^k}{\omega^2 \prod_{i=1}^{k}\omega_i^2} + \sum_{i=1}^{k} \frac{1}{\omega_i^2(\omega^2 - \omega_i^2)\prod_{j\neq i}(\omega_i^2 - \omega_j^2)} \tag{S31}$$

$$\frac{\omega^{2n}}{\prod_{i=1}^{k}(\omega^2 - \omega_i^2)} = \sum_{i=1}^{k} \frac{\omega_i^{2n}}{(\omega^2 - \omega_i^2)\prod_{j\neq i}(\omega_i^2 - \omega_j^2)} \tag{S32}$$

with $n \geq 0$ to rewrite the fraction of Eq. (S27) as a sum of terms proportional to $(\omega^2 - \omega_i^2)^{-1}$ and one term proportional to ω^{-2}. Using the solutions of the integrals

$$\int_{-\infty}^{\infty} \frac{e^{i\omega t} - 1}{\omega^2 - \omega_i^2} d\omega = \frac{\pi(e^{-\sqrt{-\omega_i^2}t} - 1)}{\sqrt{-\omega_i^2}} \tag{S33}$$

$$\int_{-\infty}^{\infty} \frac{e^{i\omega t} - 1}{\omega^2} d\omega = -\pi t \tag{S34}$$

for $t > 0$ with the condition $\mathrm{Re}(\omega_i^2) < 0 \vee \mathrm{Im}(\omega_i^2) \neq 0 \wedge \mathrm{Im}(\sqrt{\omega_i^2}) \neq 0$, we can rewrite the integral of the MSD Eq. (S27) as

$$\boxed{C_{\mathrm{MSD}}(t) = \frac{B}{c_5}\left(\frac{-k_1 t}{\omega_1^2\omega_2^2\omega_3^2\omega_4^2} + \sum_{i=1}^{4} \frac{e^{-\sqrt{-\omega_i^2}t} - 1}{\sqrt{-\omega_i^2}\prod_{j\neq i}(\omega_i^2 - \omega_j^2)}\left[\frac{k_1}{\omega_i^2} + k_2 + k_3\omega_i^2\right]\right)}\,. \tag{S35}$$

The poles, i.e. the roots of the polynomial Eq. (S29), can be determined analytically for polynomials up to fourth order, which one obtains for $n = 3$ exponentials without potential or $n = 2$ exponentials in a harmonic potential. For any larger n, the roots of the polynomials of Eq. (S25) or Eq. (S21), have to be solved numerically. We determine those roots using *sympy* in Python.

The constants c_i and k_i in Eqs. (S23) and (S25) that define the MSD via Eq. (S26) for a $n = 5$ exponential memory kernel (Eq. (S15)) without any potential are given by

$$\boxed{\begin{aligned}
c_5 =& \tau_3^2\tau_4^2\tau_5^2 + \tau_2^2(\tau_3^2\tau_4^2 + (\tau_3^2 + (1 - 2(a_2 + a_3 + a_4 + a_5)\tau_3^2)\tau_4^2)\tau_5^2) \\
&+ \tau_1^2[\tau_3^2\tau_4^2 + (\tau_4^2 + \tau_3^2(1 - 2(a_1 + a_3 + a_4 + a_5)\tau_4^2))\tau_5^2 + \tau_2^2[\tau_4^2 + (1 - 2(a_1 + a_2 + a_4 + a_5)\tau_4^2)\tau_5^2 \\
&+ \tau_3^2(1 - 2(a_1 + a_2 + a_3 + a_4)\tau_4^2 + (-2(a_1 + a_2 + a_3 + a_5) + (a_1 + a_2 + a_3 + a_4 + a_5)^2\tau_4^2)\tau_5^2)]] \\
c_6 =& \tau_2^2\tau_3^2\tau_4^2\tau_5^2 + \tau_1^2[\tau_3^2\tau_4^2\tau_5^2 + \tau_2^2(\tau_3^2\tau_4^2 + (\tau_3^2 + (1 - 2(a_1 + a_2 + a_3 + a_4 + a_5)\tau_3^2)\tau_4^2)\tau_5^2)] \\
c_7 =& \tau_1^2\tau_2^2\tau_3^2\tau_4^2\tau_5^2
\end{aligned}} \tag{S36}$$

$$
\begin{aligned}
k_1 =& -2(a_1\tau_1 + a_2\tau_2 + a_3\tau_3 + a_4\tau_4 + a_5\tau_5)\\
k_2 =& -2(a_3\tau_1^2\tau_3 + a_3\tau_2^2\tau_3 + a_4\tau_1^2\tau_4 + a_4\tau_2^2\tau_4 + a_4\tau_3^2\tau_4 + a_3\tau_3\tau_4^2 + a_5(\tau_1^2 + \tau_2^2 + \tau_3^2 + \tau_4^2)\tau_5\\
&+(a_3\tau_3 + a_4\tau_4)\tau_5^2 + a_2\tau_2(\tau_1^2 + \tau_3^2 + \tau_4^2 + \tau_5^2) + a_1\tau_1(\tau_2^2 + \tau_3^2 + \tau_4^2 + \tau_5^2))\\
k_3 =& -2[a_2\tau_1^2\tau_2\tau_3^2 + a_1\tau_1\tau_2^2\tau_3^2 + a_4\tau_1^2\tau_2^2\tau_4 + a_4\tau_1^2\tau_3^2\tau_4 + a_4\tau_2^2\tau_3^2\tau_4 + a_2\tau_1^2\tau_2\tau_4^2 + a_1\tau_1\tau_2^2\tau_4^2 + a_1\tau_1\tau_3^2\tau_4^2\\
&+a_2\tau_2\tau_3^2\tau_4^2 + a_5(\tau_3^2\tau_4^2 + \tau_2^2(\tau_3^2 + \tau_4^2) + \tau_1^2(\tau_2^2 + \tau_3^2 + \tau_4^2))\tau_5\\
&+(a_4(\tau_1^2 + \tau_2^2 + \tau_3^2)\tau_4 + a_2\tau_2(\tau_1^2 + \tau_3^2 + \tau_4^2) + a_1\tau_1(\tau_2^2 + \tau_3^2 + \tau_4^2))\tau_5^2\\
&+a_3\tau_3(\tau_4^2\tau_5^2 + \tau_2^2(\tau_4^2 + \tau_5^2) + \tau_1^2(\tau_2^2 + \tau_4^2 + \tau_5^2))]\\
k_4 =& -2(\tau_1\tau_2\tau_3\tau_4(a_4\tau_1\tau_2\tau_3 + (a_3\tau_1\tau_2 + a_2\tau_1\tau_3 + a_1\tau_2\tau_3)\tau_4) + a_5(\tau_2^2\tau_3^2\tau_4^2 + \tau_1^2(\tau_3^2\tau_4^2 + \tau_2^2(\tau_3^2 + \tau_4^2)))\tau_5\\
&+(\tau_1\tau_2\tau_3(a_3\tau_1\tau_2 + a_2\tau_1\tau_3 + a_1\tau_2\tau_3) + a_4(\tau_2^2\tau_3^2 + \tau_1^2(\tau_2^2 + \tau_3^2))\tau_4 + (a_3(\tau_1^2 + \tau_2^2)\tau_3\\
&+a_2\tau_2(\tau_1^2 + \tau_3^2) + a_1\tau_1(\tau_2^2 + \tau_3^2))\tau_4^2)\tau_5^2)\\
k_5 =& -2\tau_1\tau_2\tau_3\tau_4\tau_5(a_5\tau_1\tau_2\tau_3\tau_4 + a_4\tau_1\tau_2\tau_3\tau_5 + (a_3\tau_1\tau_2 + a_2\tau_1\tau_3 + a_1\tau_2\tau_3)\tau_4\tau_5)\\
c_1 =&(a_1\tau_1 + a_2\tau_2 + a_3\tau_3 + a_4\tau_4 + a_5\tau_5)^2\\
c_2 =&1 - 2a_2\tau_2^2 - 2a_3\tau_3^2 + a_3^2\tau_1^2\tau_3^2 + a_3^2\tau_2^2\tau_3^2 + 2a_3a_4\tau_1^2\tau_3\tau_4 + 2a_3a_4\tau_2^2\tau_3\tau_4 - 2a_4\tau_4^2 + a_4^2\tau_1^2\tau_4^2 + a_4^2\tau_2^2\tau_4^2\\
&+a_3^2\tau_3^2\tau_4^2 + 2a_3a_4\tau_3^2\tau_4^2 + a_4^2\tau_3^2\tau_4^2 + 2a_5(a_4(\tau_1^2 + \tau_2^2 + \tau_3^2)\tau_4 + a_3\tau_3(\tau_1^2 + \tau_2^2 + \tau_4^2))\tau_5\\
&+((a_3\tau_3 + a_4\tau_4)^2 + a_5^2(\tau_1^2 + \tau_2^2 + \tau_3^2 + \tau_4^2) + 2a_5(-1 + a_3\tau_3^2 + a_4\tau_4^2))\tau_5^2 + a_2^2\tau_2^2(\tau_1^2 + \tau_3^2 + \tau_4^2 + \tau_5^2)\\
&+a_1^2\tau_1^2(\tau_2^2 + \tau_3^2 + \tau_4^2 + \tau_5^2) + 2a_2\tau_2(a_5\tau_5(\tau_1^2 + \tau_3^2 + \tau_4^2 + \tau_2\tau_5) + a_4\tau_4(\tau_1^2 + \tau_3^2 + \tau_2\tau_4 + \tau_5^2)\\
&+a_3\tau_3(\tau_1^2 + \tau_2\tau_3 + \tau_4^2 + \tau_5^2)) + 2a_1\tau_1(a_2\tau_2\tau_3^2 + a_4\tau_2^2\tau_4 + a_4\tau_3^2\tau_4 + a_2\tau_2\tau_4^2 + a_5(\tau_2^2 + \tau_3^2 + \tau_4^2)\tau_5\\
&+(a_2\tau_2 + a_4\tau_4)\tau_5^2 + a_3\tau_3(\tau_2^2 + \tau_4^2 + \tau_5^2) + \tau_1(-1 + a_2\tau_2^2 + a_3\tau_3^2 + a_4\tau_4^2 + a_5\tau_5^2))\\
c_3 =&\tau_4^2 + \tau_3^2(1 - 2(a_3 + a_4)\tau_4^2) + \tau_5^2 + (-2(a_3 + a_5)\tau_3^2 + (-2(a_4 + a_5) + (a_3 + a_4 + a_5)^2\tau_3^2)\tau_4^2)\tau_5^2\\
&+2a_2\tau_2\tau_3\tau_4\tau_5(a_5\tau_3\tau_4 + a_4\tau_3\tau_5 + a_3\tau_4\tau_5) + 2a_1\tau_1[\tau_2\tau_3\tau_4(a_4\tau_2\tau_3 + a_3\tau_2\tau_4 + a_2\tau_3\tau_4)\\
&+a_5(\tau_3^2\tau_4^2 + \tau_2^2(\tau_3^2 + \tau_4^2))\tau_5 + (a_4(\tau_2^2 + \tau_3^2)\tau_4 + a_3\tau_3(\tau_2^2 + \tau_4^2) + a_2\tau_2(\tau_3^2 + \tau_4^2))\tau_5^2]\\
&+\tau_2^2[1 - 2a_4\tau_4^2 + \tau_3^2(-2a_3 + (a_3 + a_4)^2\tau_4^2) + 2a_5\tau_3\tau_4(a_4\tau_3 + a_3\tau_4)\tau_5\\
&+((a_3\tau_3 + a_4\tau_4)^2 + a_5^2(\tau_3^2 + \tau_4^2) + 2a_5(-1 + a_3\tau_3^2 + a_4\tau_4^2))\tau_5^2 + a_2^2(\tau_4^2\tau_5^2 + \tau_3^2(\tau_4^2 + \tau_5^2))\\
&+2a_2(-\tau_4^2 + (-1 + (a_4 + a_5)\tau_4^2)\tau_5^2 + \tau_3^2(-1 + (a_3 + a_4)\tau_4^2 + (a_3 + a_5)\tau_5^2))]\\
&+\tau_1^2[1 - 2a_3\tau_3^2 + a_3^2\tau_2^2\tau_3^2 + 2a_3a_4\tau_2^2\tau_3\tau_4 - 2a_4\tau_4^2 + a_4^2\tau_2^2\tau_4^2 + a_3^2\tau_3^2\tau_4^2 + 2a_3a_4\tau_3^2\tau_4^2 + a_4^2\tau_3^2\tau_4^2\\
&+2a_5(a_4(\tau_2^2 + \tau_3^2)\tau_4 + a_3\tau_3(\tau_2^2 + \tau_4^2))\tau_5\\
&+((a_3\tau_3 + a_4\tau_4)^2 + a_5^2(\tau_2^2 + \tau_3^2 + \tau_4^2) + 2a_5(-1 + a_3\tau_3^2 + a_4\tau_4^2))\tau_5^2 + a_2^2\tau_2^2(\tau_3^2 + \tau_4^2 + \tau_5^2)\\
&+a_1^2(\tau_4^2\tau_5^2 + \tau_3^2(\tau_4^2 + \tau_5^2) + \tau_2^2(\tau_3^2 + \tau_4^2 + \tau_5^2)) + 2a_2\tau_2(\tau_3\tau_4(a_4\tau_3 + a_3\tau_4) + a_5(\tau_3^2 + \tau_4^2)\tau_5\\
&+(a_3\tau_3 + a_4\tau_4)\tau_5^2 + \tau_2(-1 + a_3\tau_3^2 + a_4\tau_4^2 + a_5\tau_5^2)) + 2a_1(-\tau_4^2 + (-1 + (a_4 + a_5)\tau_4^2)\tau_5^2\\
&+\tau_3^2(-1 + (a_3 + a_4)\tau_4^2 + (a_3 + a_5)\tau_5^2) + \tau_2^2(-1 + a_3\tau_3^2 + a_4\tau_4^2 + a_5\tau_5^2 + a_2(\tau_3^2 + \tau_4^2 + \tau_5^2)))]\\
c_4 =&\tau_3^2\tau_4^2 + (\tau_4^2 + \tau_3^2(1 - 2(a_3 + a_4 + a_5)\tau_4^2))\tau_5^2 + 2a_1\tau_1\tau_2\tau_3\tau_4\tau_5(a_5\tau_2\tau_3\tau_4\\
&+(a_4\tau_2\tau_3 + a_3\tau_2\tau_4 + a_2\tau_3\tau_4)\tau_5)\\
&+\tau_2^2(\tau_5^2 + \tau_4^2(1 - 2(a_2 + a_4 + a_5)\tau_5^2) + \tau_3^2(1 - 2(a_2 + a_3 + a_4)\tau_4^2\\
&+(-2(a_2 + a_3 + a_5) + (a_2 + a_3 + a_4 + a_5)^2\tau_4^2)\tau_5^2)) + \tau_1^2[\tau_3^2 + \tau_4^2 - 2(a_1 + a_3 + a_4)\tau_3^2\tau_4^2\\
&+(1 - 2(a_1 + a_3 + a_5)\tau_3^2 + (-2(a_1 + a_4 + a_5) + (a_1 + a_3 + a_4 + a_5)^2\tau_3^2)\tau_4^2)\tau_5^2\\
&+2a_2\tau_2\tau_3\tau_4\tau_5(a_5\tau_3\tau_4 + a_4\tau_3\tau_5 + a_3\tau_4\tau_5) + \tau_2^2[1 - 2a_4\tau_4^2 + \tau_3^2(-2a_3 + (a_3 + a_4)^2\tau_4^2)\\
&+2a_5\tau_3\tau_4(a_4\tau_3 + a_3\tau_4)\tau_5 + ((a_3\tau_3 + a_4\tau_4)^2 + a_5^2(\tau_3^2 + \tau_4^2) + 2a_5(-1 + a_3\tau_3^2 + a_4\tau_4^2))\tau_5^2\\
&+a_1^2(\tau_4^2\tau_5^2 + \tau_3^2(\tau_4^2 + \tau_5^2)) + a_2^2(\tau_4^2\tau_5^2 + \tau_3^2(\tau_4^2 + \tau_5^2))\\
&+2a_2(-\tau_4^2 + (-1 + (a_4 + a_5)\tau_4^2)\tau_5^2 + \tau_3^2(-1 + (a_3 + a_4)\tau_4^2 + (a_3 + a_5)\tau_5^2))\\
&+2a_1(-\tau_4^2 + (-1 + (a_2 + a_4 + a_5)\tau_4^2)\tau_5^2 + \tau_3^2(-1 + (a_2 + a_3 + a_4)\tau_4^2 + (a_2 + a_3 + a_5)\tau_5^2))]]\,.
\end{aligned}
$$

For $n = 3$ exponentials in a harmonic potential, the constants of Eqs. (S17) and (S21) are given by

$$\boxed{\begin{aligned}
k_1 =& -2(a\tau_1 + b\tau_2 + c\tau_3) \\
k_2 =& -2(b\tau_1^2\tau_2 + a\tau_1\tau_2^2 + c\tau_1^2\tau_3 + c\tau_2^2\tau_3 + a\tau_1\tau_3^2 + b\tau_2\tau_3^2) \\
k_3 =& -2\tau_1\tau_2\tau_3(c\tau_1\tau_2 + b\tau_1\tau_3 + a\tau_2\tau_3) \\
c_0 =& K^2 \\
c_1 =& (a\tau_1 + b\tau_2 + c\tau_3)^2 + K^2(\tau_1^2 + \tau_2^2 + \tau_3^2) + 2K(-1 + a\tau_1^2 + b\tau_2^2 + c\tau_3^2) \\
c_2 =& 1 - 2K\tau_1^2 - 2b\tau_2^2 - 2K\tau_2^2 + b^2\tau_1^2\tau_2^2 + 2bK\tau_1^2\tau_2^2 + K^2\tau_1^2\tau_2^2 + 2bc\tau_1^2\tau_2\tau_3 \\
& + (-2c - 2K + c^2\tau_1^2 + 2cK\tau_1^2 + K^2\tau_1^2 + (b + c + K)^2\tau_2^2)\tau_3^2 \\
& + a^2\tau_1^2(\tau_2^2 + \tau_3^2) + 2a\tau_1(\tau_1(-1 + (b + K)\tau_2^2) + c\tau_2^2\tau_3 + ((c + K)\tau_1 + b\tau_2)\tau_3^2) \\
c_3 =& \tau_3^2 + \tau_2^2(1 - 2(b + c + K)\tau_3^2) + \tau_1^2(1 - 2(a + b + K)\tau_2^2 \\
& + (-2(a + c + K) + (a + b + c + K)^2\tau_2^2)\tau_3^2) \\
c_4 =& \tau_2^2\tau_3^2 + \tau_1^2(\tau_3^2 + \tau_2^2(1 - 2(a + b + c + K)\tau_3^2)) \\
c_5 =& \tau_1^2\tau_2^2\tau_3^2
\end{aligned}} \tag{S39}$$

For $n=5$ exponentials in a harmonic potential, the constants of Eqs. (S17) and (S21) are given by

$$
\begin{aligned}
c_4 =&\tau_3^2\tau_4^2+(\tau_4^2+\tau_3^2(1-2(a_3+a_4+a_5+K)\tau_4^2))\tau_5^2+2a_1\tau_1\tau_2\tau_3\tau_4\tau_5(a_5\tau_2\tau_3\tau_4\\
&+(a_4\tau_2\tau_3+a_3\tau_2\tau_4+a_2\tau_3\tau_4)\tau_5)\\
&+\tau_2^2(\tau_4^2+(1-2(a_2+a_4+a_5+K)\tau_4^2)\tau_5^2+\tau_3^2(1-2(a_2+a_3+a_4+K)\tau_4^2+(-2(a_2+a_3+a_5+K)\\
&+(a_2+a_3+a_4+a_5+K)^2\tau_4^2)\tau_5^2))\\
&+\tau_1^2(\tau_3^2+\tau_4^2-2(a_1+a_3+a_4+K)\tau_3^2\tau_4^2+(1-2(a_1+a_3+a_5+K)\tau_3^2\\
&+(-2(a_1+a_4+a_5+K)+(a_1+a_3+a_4+a_5+K)^2\tau_3^2)\tau_4^2)\tau_5^2\\
&+2a_2\tau_2\tau_3\tau_4\tau_5(a_5\tau_3\tau_4+a_4\tau_3\tau_5+a_3\tau_4\tau_5)\\
&+\tau_2^2[1-2a_1\tau_3^2-2a_2\tau_3^2-2a_3\tau_3^2-2K\tau_3^2-2a_1\tau_4^2-2a_2\tau_4^2-2a_4\tau_4^2-2K\tau_4^2+a_1^2\tau_3^2\tau_4^2+2a_1a_2\tau_3^2\tau_4^2\\
&+a_2^2\tau_3^2\tau_4^2+2a_1a_3\tau_3^2\tau_4^2+2a_2a_3\tau_3^2\tau_4^2+a_3^2\tau_3^2\tau_4^2+2a_1a_4\tau_3^2\tau_4^2\\
&+2a_2a_4\tau_3^2\tau_4^2+2a_3a_4\tau_3^2\tau_4^2+a_4^2\tau_3^2\tau_4^2+2a_1K\tau_3^2\tau_4^2\\
&+2a_2K\tau_3^2\tau_4^2+2a_3K\tau_3^2\tau_4^2+2a_4K\tau_3^2\tau_4^2+K^2\tau_3^2\tau_4^2\\
&+2a_5\tau_3\tau_4(a_4\tau_3+a_3\tau_4)\tau_5+(-2(a_2+a_5+K)+(a_2+a_3+a_5+K)^2\tau_3^2+2a_3a_4\tau_3\tau_4\\
&+(a_2+a_4+a_5+K)^2\tau_4^2+a_1^2(\tau_3^2+\tau_4^2)\\
&+2a_1(-1+(a_2+a_3+a_5+K)\tau_3^2+(a_2+a_4+a_5+K)\tau_4^2))\tau_5^2])\\
c_5 =&\tau_3^2\tau_4^2\tau_5^2+\tau_2^2(\tau_3^2\tau_4^2+(\tau_3^2+(1-2(a_2+a_3+a_4+a_5+K)\tau_3^2)\tau_4^2)\tau_5^2)\\
&+\tau_1^2(\tau_3^2\tau_4^2+(\tau_4^2+\tau_3^2(1-2(a_1+a_3+a_4+a_5+K)\tau_4^2))\tau_5^2\\
&+\tau_2^2(\tau_4^2+(1-2(a_1+a_2+a_4+a_5+K)\tau_4^2)\tau_5^2\\
&+\tau_3^2(1-2(a_1+a_2+a_3+a_4+K)\tau_4^2+(-2(a_1+a_2+a_3+a_5+K)\\
&+(a_1+a_2+a_3+a_4+a_5+K)^2\tau_4^2)\tau_5^2)))\\
c_6 =&\tau_2^2\tau_3^2\tau_4^2\tau_5^2+\tau_1^2(\tau_3^2\tau_4^2\tau_5^2+\tau_2^2(\tau_3^2\tau_4^2+(\tau_3^2+(1-2(a_1+a_2+a_3+a_4+a_5+K)\tau_3^2)\tau_4^2)\tau_5^2))\\
c_7 =&\tau_1^2\tau_2^2\tau_3^2\tau_4^2\tau_5^2
\end{aligned}
\tag{S40}
$$

$$
\begin{aligned}
k_1 =& -2(a_1\tau_1 + a_2\tau_2 + a_3\tau_3 + a_4\tau_4 + a_5\tau_5)\\
k_2 =& -2(a_3\tau_1^2\tau_3 + a_3\tau_2^2\tau_3 + a_4\tau_1^2\tau_4 + a_4\tau_2^2\tau_4\\
&+a_4\tau_3^2\tau_4 + a_3\tau_3\tau_4^2 + a_5(\tau_1^2 + \tau_2^2 + \tau_3^2 + \tau_4^2)\tau_5 + (a_3\tau_3 + a_4\tau_4)\tau_5^2\\
&+a_2\tau_2(\tau_1^2 + \tau_3^2 + \tau_4^2 + \tau_5^2) + a_1\tau_1(\tau_2^2 + \tau_3^2 + \tau_4^2 + \tau_5^2))\\
k_3 =& -2(a_2\tau_1^2\tau_2\tau_3^2 + a_1\tau_1\tau_2^2\tau_3^2 + a_4\tau_1^2\tau_2^2\tau_4 + a_4\tau_1^2\tau_3^2\tau_4\\
&+a_4\tau_2^2\tau_3^2\tau_4 + a_2\tau_1^2\tau_2\tau_4^2 + a_1\tau_1\tau_2^2\tau_4^2 + a_1\tau_1\tau_3^2\tau_4^2 + a_2\tau_2\tau_3^2\tau_4^2\\
&+a_5(\tau_3^2\tau_4^2 + \tau_2^2(\tau_3^2 + \tau_4^2) + \tau_1^2(\tau_2^2 + \tau_3^2 + \tau_4^2))\tau_5 + (a_4(\tau_1^2 + \tau_2^2 + \tau_3^2)\tau_4 + a_2\tau_2(\tau_1^2 + \tau_3^2 + \tau_4^2)\\
&+a_1\tau_1(\tau_2^2 + \tau_3^2 + \tau_4^2))\tau_5^2 + a_3\tau_3(\tau_4^2\tau_5^2 + \tau_2^2(\tau_4^2 + \tau_5^2) + \tau_1^2(\tau_2^2 + \tau_4^2 + \tau_5^2)))\\
k_4 =& -2\tau_1\tau_2\tau_3\tau_4(a_4\tau_1\tau_2\tau_3 + (a_3\tau_1\tau_2 + a_2\tau_1\tau_3 + a_1\tau_2\tau_3)\tau_4) - 2a_5(\tau_2^2\tau_3^2\tau_4^2 + \tau_1^2(\tau_3^2\tau_4^2 + \tau_2^2(\tau_3^2 + \tau_4^2)))\tau_5\\
&-2(\tau_1\tau_2\tau_3(a_3\tau_1\tau_2 + a_2\tau_1\tau_3 + a_1\tau_2\tau_3) + a_4(\tau_2^2\tau_3^2 + \tau_1^2(\tau_2^2 + \tau_3^2))\tau_4 + (a_3(\tau_1^2 + \tau_2^2)\tau_3 + a_2\tau_2(\tau_1^2 + \tau_3^2)\\
&+a_1\tau_1(\tau_2^2 + \tau_3^2))\tau_4^2)\tau_5^2\\
k_5 =& -2\tau_1\tau_2\tau_3\tau_4\tau_5(a_5\tau_1\tau_2\tau_3\tau_4 + a_4\tau_1\tau_2\tau_3\tau_5 + a_3\tau_1\tau_2\tau_4\tau_5 + a_2\tau_1\tau_3\tau_4\tau_5 + a_1\tau_2\tau_3\tau_4\tau_5)\\
c_0 =& K^2\\
c_1 =& (a_1\tau_1 + a_2\tau_2 + a_3\tau_3 + a_4\tau_4 + a_5\tau_5)^2\\
&+K^2(\tau_1^2 + \tau_2^2 + \tau_3^2 + \tau_4^2 + \tau_5^2) + 2K(-1 + a_1\tau_1^2 + a_2\tau_2^2 + a_3\tau_3^2 + a_4\tau_4^2 + a_5\tau_5^2)\\
c_2 =& 1 - 2a_2\tau_2^2 + a_2^2\tau_1^2\tau_2^2 + 2a_2a_3\tau_1^2\tau_2\tau_3 - 2a_3\tau_3^2 + a_3^2\tau_1^2\tau_3^2 + a_2^2\tau_2^2\tau_3^2 + 2a_2a_3\tau_2^2\tau_3^2 + a_3^2\tau_2^2\tau_3^2\\
&+2a_2a_4\tau_1^2\tau_2\tau_4 + 2a_3a_4\tau_1^2\tau_3\tau_4 + 2a_3a_4\tau_2^2\tau_3\tau_4 + 2a_2a_4\tau_2\tau_3^2\tau_4 - 2a_4\tau_4^2 + a_4^2\tau_1^2\tau_4^2 + a_2^2\tau_2^2\tau_4^2\\
&+2a_2a_4\tau_2^2\tau_4^2 + a_4^2\tau_2^2\tau_4^2 + 2a_2a_3\tau_2\tau_3\tau_4^2 + a_3^2\tau_3^2\tau_4^2 + 2a_3a_4\tau_3^2\tau_4^2 + a_4^2\tau_3^2\tau_4^2 + 2a_5(a_4(\tau_1^2 + \tau_2^2 + \tau_3^2)\tau_4\\
&+a_3\tau_3(\tau_1^2 + \tau_2^2 + \tau_4^2) + a_2\tau_2(\tau_1^2 + \tau_3^2 + \tau_4^2))\tau_5 + ((a_2\tau_2 + a_3\tau_3 + a_4\tau_4)^2\\
&+a_5^2(\tau_1^2 + \tau_2^2 + \tau_3^2 + \tau_4^2) + 2a_5(-1 + a_2\tau_2^2 + a_3\tau_3^2 + a_4\tau_4^2))\tau_5^2 + a_1^2\tau_1^2(\tau_2^2 + \tau_3^2 + \tau_4^2 + \tau_5^2) + K^2(\tau_3^2\tau_4^2\\
&+(\tau_3^2 + \tau_4^2)\tau_5^2 + \tau_2^2(\tau_3^2 + \tau_4^2 + \tau_5^2) + \tau_1^2(\tau_2^2 + \tau_3^2 + \tau_4^2 + \tau_5^2)) + 2K(-\tau_4^2 + \tau_3^2(-1 + (a_3 + a_4)\tau_4^2)\\
&-\tau_5^2 + ((a_3 + a_5)\tau_3^2 + (a_4 + a_5)\tau_4^2)\tau_5^2 + \tau_1^2(-1 + a_2\tau_2^2 + a_3\tau_3^2 + a_4\tau_4^2 + a_5\tau_5^2)\\
&+\tau_2^2(-1 + a_2\tau_3^2 + a_3\tau_3^2 + a_2\tau_4^2 + a_4\tau_4^2 + (a_2 + a_5)\tau_5^2)) + 2a_1\tau_1(a_2\tau_2\tau_3^2 + a_4\tau_2^2\tau_4 + a_4\tau_3^2\tau_4 + a_2\tau_2\tau_4^2\\
&+a_5(\tau_2^2 + \tau_3^2 + \tau_4^2)\tau_5 + (a_2\tau_2 + a_4\tau_4)\tau_5^2 + a_3\tau_3(\tau_2^2 + \tau_4^2 + \tau_5^2)\\
&+\tau_1(-1 + a_2\tau_2^2 + a_3\tau_3^2 + a_4\tau_4^2 + a_5\tau_5^2 + K(\tau_2^2 + \tau_3^2 + \tau_4^2 + \tau_5^2)))\\
c_3 =& \tau_4^2 + \tau_3^2(1 - 2(a_3 + a_4 + K)\tau_4^2) + \tau_5^2 + (-2(a_3 + a_5 + K)\tau_3^2 + (-2(a_4 + a_5 + K)\\
&+(a_3 + a_4 + a_5 + K)^2\tau_3^2)\tau_4^2)\tau_5^2 + 2a_2\tau_2\tau_3\tau_4\tau_5(a_5\tau_3\tau_4 + a_4\tau_3\tau_5 + a_3\tau_4\tau_5)\\
&+2a_1\tau_1(\tau_2\tau_3\tau_4(a_4\tau_2\tau_3 + a_3\tau_2\tau_4 + a_2\tau_3\tau_4) + a_5(\tau_3^2\tau_4^2 + \tau_2^2(\tau_3^2 + \tau_4^2))\tau_5 + (a_4(\tau_2^2 + \tau_3^2)\tau_4\\
&+a_3\tau_3(\tau_2^2 + \tau_4^2) + a_2\tau_2(\tau_3^2 + \tau_4^2))\tau_5^2) + \tau_2^2(1 - 2K\tau_3^2 - 2a_4\tau_4^2 - 2K\tau_4^2 + a_4^2\tau_3^2\tau_4^2\\
&+2a_4K\tau_3^2\tau_4^2 + K^2\tau_3^2\tau_4^2 + 2a_4a_5\tau_3^2\tau_4\tau_5 + (-2a_5 - 2K + a_5^2\tau_3^2 + 2a_5K\tau_3^2\\
&+K^2\tau_3^2 + (a_4 + a_5 + K)^2\tau_4^2)\tau_5^2 + a_3^2\tau_3^2(\tau_4^2 + \tau_5^2) + 2a_3\tau_3(\tau_3(-1 + (a_4 + K)\tau_4^2)\\
&+a_5\tau_4^2\tau_5 + ((a_5 + K)\tau_3 + a_4\tau_4)\tau_5^2) + a_2^2(\tau_4^2\tau_5^2 + \tau_3^2(\tau_4^2 + \tau_5^2)) + 2a_2(-\tau_4^2 + (-1 + (a_4 + a_5 + K)\tau_4^2)\tau_5^2\\
&+\tau_3^2(-1 + (a_3 + a_4 + K)\tau_4^2 + (a_3 + a_5 + K)\tau_5^2))) + \tau_1^2(1 - 2K\tau_2^2 - 2a_3\tau_3^2\\
&-2K\tau_3^2 + a_3^2\tau_2^2\tau_3^2 + 2a_3K\tau_2^2\tau_3^2 + K^2\tau_2^2\tau_3^2 + 2a_3a_4\tau_2^2\tau_3\tau_4\\
&-2a_4\tau_4^2 - 2K\tau_4^2 + a_4^2\tau_2^2\tau_4^2 + 2a_4K\tau_2^2\tau_4^2 + K^2\tau_2^2\tau_4^2 + a_3^2\tau_3^2\tau_4^2 + 2a_3a_4\tau_3^2\tau_4^2 + a_4^2\tau_3^2\tau_4^2\\
&+2a_3K\tau_3^2\tau_4^2 + 2a_4K\tau_3^2\tau_4^2 + K^2\tau_3^2\tau_4^2 + 2a_5(a_4(\tau_2^2 + \tau_3^2)\tau_4 + a_3\tau_3(\tau_2^2 + \tau_4^2))\tau_5 + ((a_3\tau_3 + a_4\tau_4)^2\\
&+a_5^2(\tau_2^2 + \tau_3^2 + \tau_4^2) + K^2(\tau_2^2 + \tau_3^2 + \tau_4^2) + 2K(-1 + a_3\tau_3^2 + a_4\tau_4^2) + 2a_5(-1 + a_3\tau_3^2 + a_4\tau_4^2\\
&+K(\tau_2^2 + \tau_3^2 + \tau_4^2)))\tau_5^2 + a_2^2\tau_2^2(\tau_3^2 + \tau_4^2 + \tau_5^2) + a_1^2(\tau_4^2\tau_5^2 + \tau_3^2(\tau_4^2 + \tau_5^2) + \tau_2^2(\tau_3^2 + \tau_4^2 + \tau_5^2))\\
&+2a_2\tau_2(\tau_3\tau_4(a_4\tau_3 + a_3\tau_4)\\
&+a_5(\tau_3^2 + \tau_4^2)\tau_5 + (a_3\tau_3 + a_4\tau_4)\tau_5^2 + \tau_2(-1 + a_3\tau_3^2 + K\tau_3^2 + a_4\tau_4^2 + K\tau_4^2 + (a_5 + K)\tau_5^2)) + 2a_1(-\tau_4^2\\
&+(-1 + (a_4 + a_5 + K)\tau_4^2)\tau_5^2 + \tau_2^2(-1 + a_2\tau_3^2 + a_3\tau_3^2 + K\tau_3^2 + a_2\tau_4^2 + a_4\tau_4^2 + K\tau_4^2\\
&+(a_2 + a_5 + K)\tau_5^2) + \tau_3^2(-1 + (a_3 + a_4 + K)\tau_4^2 + (a_3 + a_5 + K)\tau_5^2)))\,.
\end{aligned}
$$

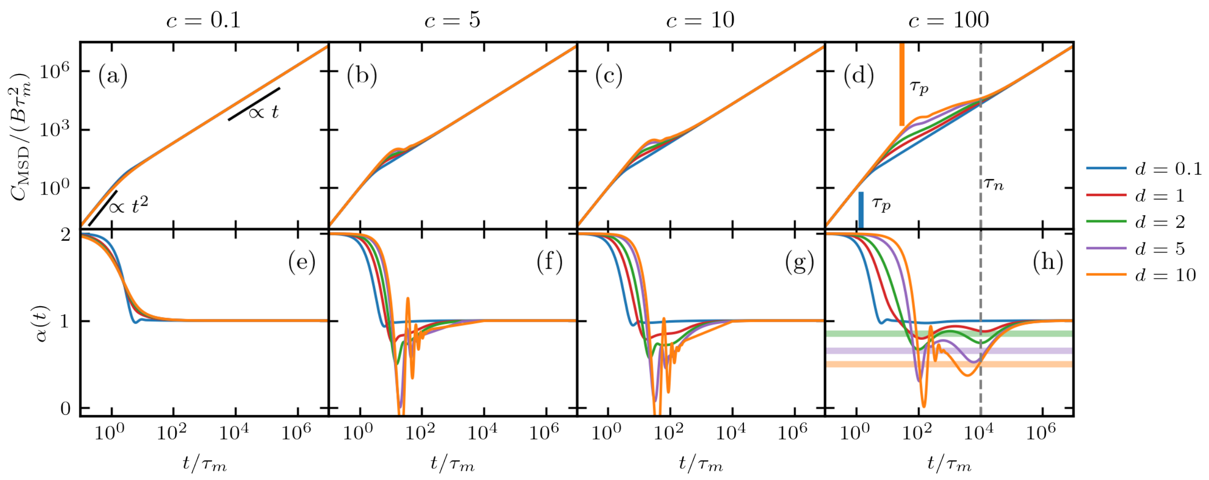

Fig. S2 Analytical MSDs from Eq. (S23) with $U(x) = 0$ for different combinations of c and d, defined in Eq. (5) with $n = 3$ exponential memory terms in the upper row and $\alpha(t)$ in the lower row. The first memory time scale is $\tau_1 = \tau_m$ and the black lines in (a) indicate the limiting exponents $\alpha = 1$ and $\alpha = 2$. The vertical lines in (d) represent the persistence time τ_p given by Eq. (11) in the main text. The thick horizontal lines in (g) and (h) represent the prediction of subdiffusive scaling α by Eq. (S8) in the respective color.

III Subfiffusion with different c and n

In Fig. 2 of the main text, we show the MSD and its time-dependent exponent $\alpha(t)$ for $n = 5$ exponentials in the memory kernel Eq. (S15). The subdiffusive regime, resulting from certain combinations of friction amplitudes and time scales defined via Eq. (5), is prolonged by using more exponential memory components, since the longest memory time τ_n increases, as indicated by the end of the subdiffusive regime at τ_n in Fig. S2d,h and in the main text Fig. 2c,f,i. The running integral $G(t)$ can be used to predict the persistence time τ_p, as discussed in the main text. We determine τ_p by iterating $\tau_j^p = 1/G(\tau_{j-1}^p)$ until reaching a fixed point; this can be done independently of whether $G(t)$ is extracted from data or whether one uses an analytical expression.

Minima of $\alpha(t)$ can occur separately if the ratio of the temporal scales c becomes large, as demonstrated in Fig. S3 for $n = 3$. One can see an intermediate diffusive regime between the subdiffusive regimes in Fig. S3a, which exhibits a larger diffusivity for higher values of d, i.e., with less momentary friction $G(t)$ on this time scale. The diffusivity of this intermediate regime is determined by the intermediate plateau of the integrated friction $G(t)$, seen in Fig. S4b. The corresponding time-dependent exponent $\alpha(t)$ in Fig. S3b exhibits two distinct minima separated by an intermediate diffusive regime with $\alpha = 1$. For large separation of the memory times, the shortest memory time acts similarly to a delta-contribution at time zero. The friction amplitude of this shortest memory contribution compared to the total friction, $\gamma_1/G(\infty)$, decreases as d increases, as seen by decreasing intermediate plateau values in $G(t)$ in Fig. S4b, which leads to increasingly long ballistic regimes for increasing d. For $d = 1$, the end of the ballistic regime and the first minimum in $\alpha(t)$ are well separated so that there is another diffusive regime in between, meaning a total of three diffusive regimes with $\alpha = 1$, as seen for the blue line in Fig. S3b.

IV Simulation details

The MSD curves in Fig. S2 are obtained by our analytical result, derived in Sec. II. However, for the MSDs in a double well potential, shown in the main text Figs. 5 and 6, results are obtained by averaging over 1000 independent simulation trajectories, each of length 10^8 time steps, where we first perform a time average to obtain the MSD for a single trajectory and then average over the trajectory ensemble. The time steps in the simulations are given for Fig. 5a and b as $\Delta = 0.1\tau_m$ and $\Delta = \tau_m$, respectively. Here, the simulations are performed by solving Eq. (9) with a fourth-order Runge-Kutta integrator, and initial conditions for each simulation are drawn from the respective Boltzmann distribution. In Fig. 6, the simulation time step is $\Delta = 100\tau_m$, and we use the Runge-Kutta integrator to solve the Markovian embedding that describes the GLE Eq. (2) with the kernel Eq. (4) [39, 61]. As a test of the simulation accuracy, we compare the MSD from a simulation in a harmonic potential to our analytical prediction Eq. (S22) in Fig. S5. We show the time averaged MSD of a single trajectory, the time and ensemble averaged MSD from ten trajectories and

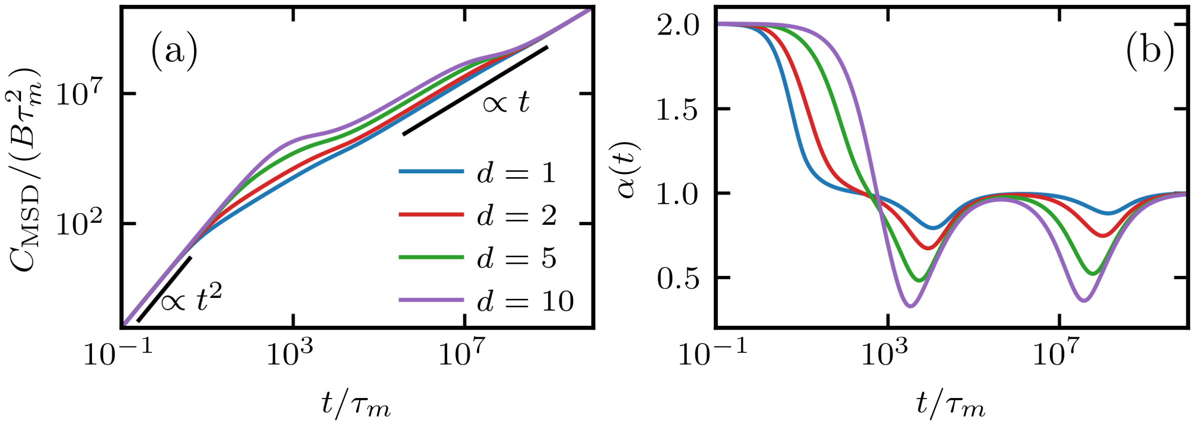

Fig. S3 (a) Analytical MSDs according to Eq. (S23) without external potential, $U(x) = 0$ for $n = 3$, $c = 10^4$ and $\tau_1 = \tau_m$ and (b) the respective time-dependent exponent $\alpha(t)$. Black lines in the MSD indicate the asymptotic scaling behavior.

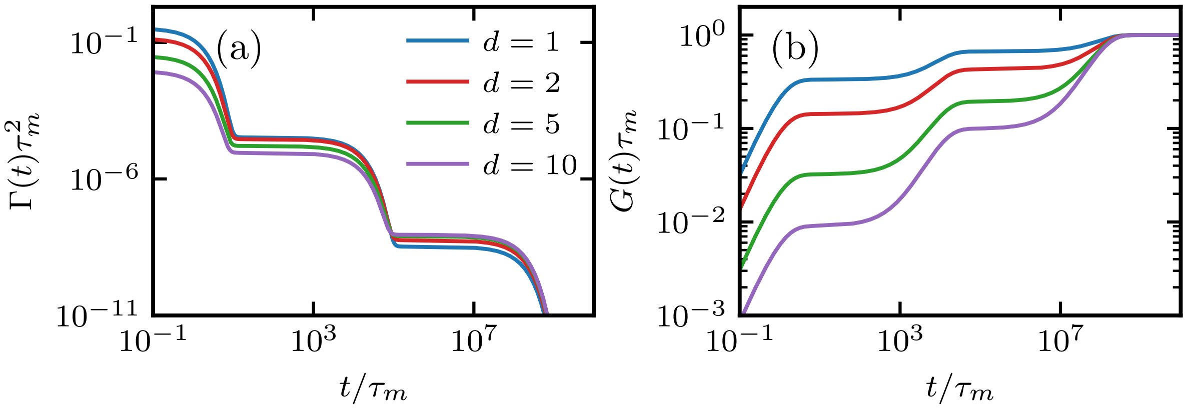

Fig. S4 (a) Multi-exponential kernel $\Gamma(t)$ for $n = 3$, $c = 10^4$ and $\tau_1 = \tau_m$ and (b) the respective running integral $G(t)$.

from 1000 trajectories. As expected, the MSD is less noisy when more data is used for averaging, i.e. at short times and when averaging over many trajectories. The excellent agreement between simulation and theory confirms the applicability of our simulation setup to describe the GLE dynamics.

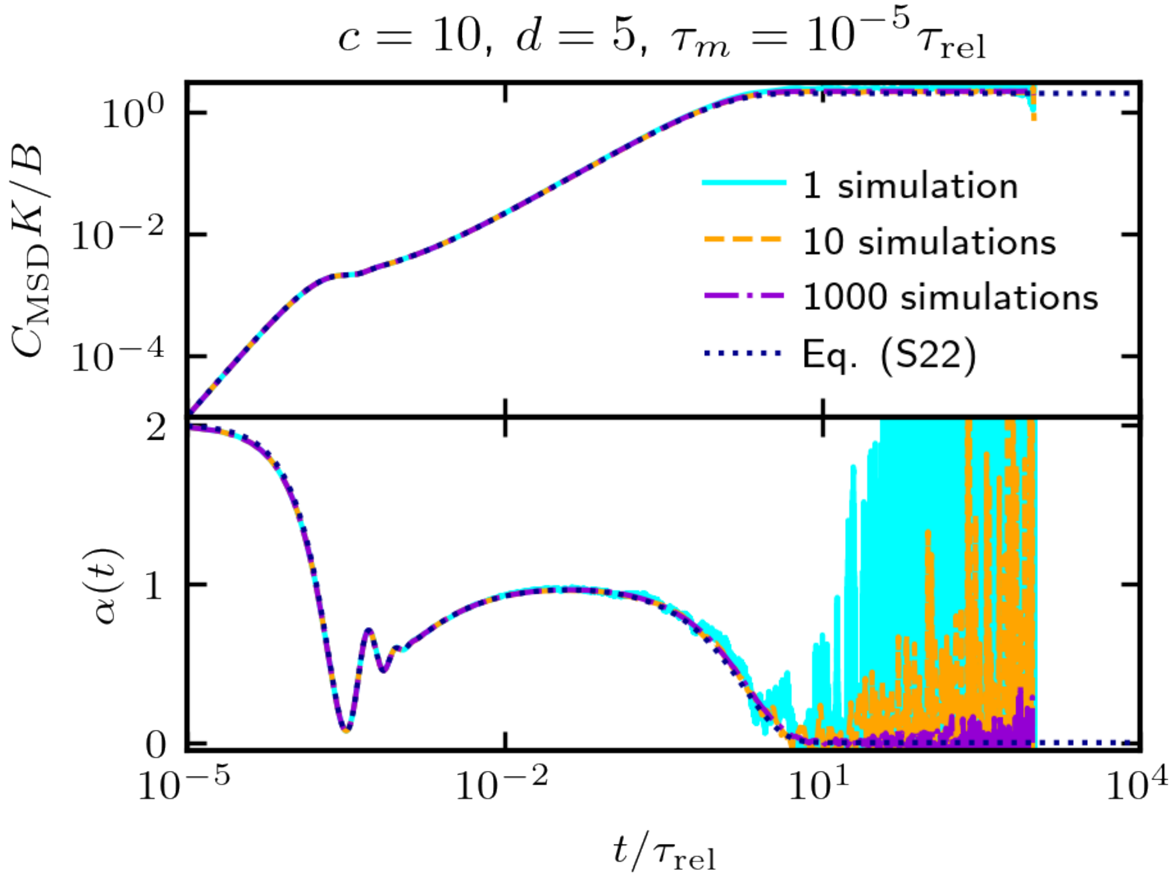

Fig. S5 MSD and $\alpha(t)$ from simulations in a harmonic potential are compared to the analytical result of Eq. (S22). The MSD from simulation results is first time averaged and then averaged over the number of trajectories given in the legend. The kernel parameters are $n = 3$, $c = 10$, $d = 5$, $\tau_m = 10^{-5}\tau_{\rm rel}$ and $\tau_1 = \tau_m$, as for the dark-blue curves in the main text in Fig. 4b,d.
